# Discovery of new acetamide derivatives of 5-indole-1,3,4-oxadiazol-2-thiol as inhibitors of HIV-1 Tat-mediated viral transcription

**DOI:** 10.1128/aac.00643-24

**Published:** 2024-09-04

**Authors:** YoungHyun Shin, Chul Min Park, Dong-Eun Kim, Sungmin Kim, Sang-Yeop Lee, Jun Young Lee, Won-Hui Jeon, Hong Gi Kim, Songmee Bae, Cheol-Hee Yoon

**Affiliations:** 1 Division of Chronic Viral Diseases, Center for Emerging Virus Research, Korea National Institute of Health, Cheongju, Republic of Korea; 2 Infectious Diseases Therapeutic Research Center, Korea Research Institute of Chemical Technology, Daejeon, Republic of Korea; 3 Medicinal Chemistry and Pharmacology, Korea University of Science and Technology, Daejeon, Republic of Korea; 4 Research Center for Bioconvergence Analysis, Ochang Center, Korea Basic Science Institute, Cheongju-si, Republic of Korea; IrsiCaixa Institut de Recerca de la Sida, Barcelona, Spain

**Keywords:** human immunodeficiency virus-1, Tat inhibitor, anti-retroviral drugs

## Abstract

Human immunodeficiency virus-1 (HIV-1) encodes a transcriptional factor called Tat, which is critical for viral transcription. Tat-mediated transcription is highly ordered apart from the cellular manner; therefore, it is considered a target for developing anti-HIV-1 drugs. However, drugs targeting Tat-mediated viral transcription are not yet available. Our high-throughput screen of a compound library employing a dual-reporter assay identified a 1,3,4-oxadiazole scaffold against Tat and HIV-1 infection. Furthermore, a serial structure–activity relation (SAR) study performed with biological assays found 1,3,4-oxadiazole derivatives (**9** and **13**) containing indole and acetamide that exhibited potent inhibitory effects on HIV-1 infectivity, with half-maximal effective concentrations (EC_50_) of 0.17 (**9**) and 0.24 µM (**13**), respectively. The prominent derivatives specifically interfered with the viral transcriptional step without targeting other infection step(s) and efficiently inhibited the HIV-1 replication cycle in the T cell lines and peripheral blood mononuclear cells infected with HIV-1. Additionally, compared to the wild type, the compounds exhibited similar potency against anti-retroviral drug-resistant HIV-1 strains. In a series of mode-of-action studies, the compounds inhibited the ejection of histone H3 for facilitating viral transcription on the long-terminal repeat (LTR) promoter. Furthermore, SAHA (a histone deacetylase inhibitor) treatment abolished the compound potency, revealing that the compounds can possibly target Tat-regulated epigenetic modulation of LTR to inhibit viral transcription. Overall, our screening identified novel 1,3,4-oxadiazole compounds that inhibited HIV-1 Tat, and subsequent SAR-based optimization led to the derivatives **9** and **13** development that could be a promising scaffold for developing a new class of therapeutic agents for HIV-1 infection.

## INTRODUCTION

Acquired immunodeficiency syndrome (AIDS) is caused by CD4^+^ T cell depletion infected with human immunodeficiency virus-1 (HIV-1) that is harmful to public health worldwide (1, 2). Currently, at least six classes of anti-retroviral drugs (ARVs) have been developed to treat individuals with HIV-1 that target specific steps of the viral life cycle (3). Although combinational anti-retroviral therapy (cART) is successful in suppressing viral load and extending the life span of HIV-1-infected individuals, rising issues exist that relate to the adverse side effects, drug-resistant mutations, and poor compliance caused by long-term cART treatment (4, 5). Therefore, developing new therapeutic agents to treat HIV-1 infection is still desired.

Tat facilitates the elongation of HIV-1 gene transcription, which is essential in the viral replication cycle. Transcription by Tat is distinguishable from that by the host cells and involves highly ordered processes such as the access of Tat to transactivating response element (TAR) RNA on the integrated long-terminal repeat (LTR) promoter, subsequent positive transcriptional elongation factor b (composed of cyclin T1 and cyclin-dependent kinase 9) recruitment of Tat, followed by hyper-phosphorylation of the C-terminal domain of RNA polymerase II (pol II), leading to transcriptional elongation enhancement (6, 7). Therefore, Tat-mediated viral transcription is considered a target to discover potent therapeutic agents against HIV-1 infection (8–10). Numerous approaches using biochemistry/biophysics or LTR promoter assays have attempted to identify the potent inhibitory compound of Tat-dependent HIV-1 transcription. However, none of the inhibitors targeting Tat-mediated viral transcription have been commercially available for treating individuals infected with HIV-1 because of their potent off-targeted effect. Currently used LTR promoter-driven reporter assays combined with transient transfection might be inefficient in screening a wide range of compounds, being difficult to distinguish the Tat-specific inhibition from the off-target effect merged with cellular toxicity. Therefore, we recently developed a cellular screening system capable of simultaneously evaluating the functional activities of Tat-induced LTR transcription and general cellular expression. The screening system distinguished the inhibitory effect on Tat-mediated transcription from that on the transcription/translation of the host cell (11, 12).

Small heterocyclic molecules that are crucial for biological activities are extensively studied in drug design. In this context, oxadiazole contains a five-membered aromatic ring and has provided privileged templates for medicinal chemistry considering that their derivatives have a wide range of pharmacological activities (anti-diabetic, antitubercular, anti-cancer, anti-fungal, anti-inflammatory, and antibacterial) (13–15). Recently, several compounds possessing an oxadiazole component exhibited potent inhibitory effects against HIV-1 infection, targeting viral enzymatic activities, such as integrase (16, 17), protease (18), and reverse-transcriptase (19–21). Despite its versatile biological activities, none of the oxadiazole derivatives are proposed as a compound against the Tat activity of HIV-1.

Herein, we identified and optimized novel HIV-1 Tat inhibitory compounds that contain an oxadiazole core. In the cells infected with wild-type and ARV-resistant HIV-1 strains, the inhibitory effects of these oxadiazole compounds on HIV-1 infectivity and replication cycle were evaluated. Furthermore, we explored the possible modes of action of these compounds underlying the inhibition of Tat-mediated viral transcription.

## RESULTS

### Identification of an oxadiazole derivative targeting HIV-1 Tat-mediated transcription

**Fig 1:**
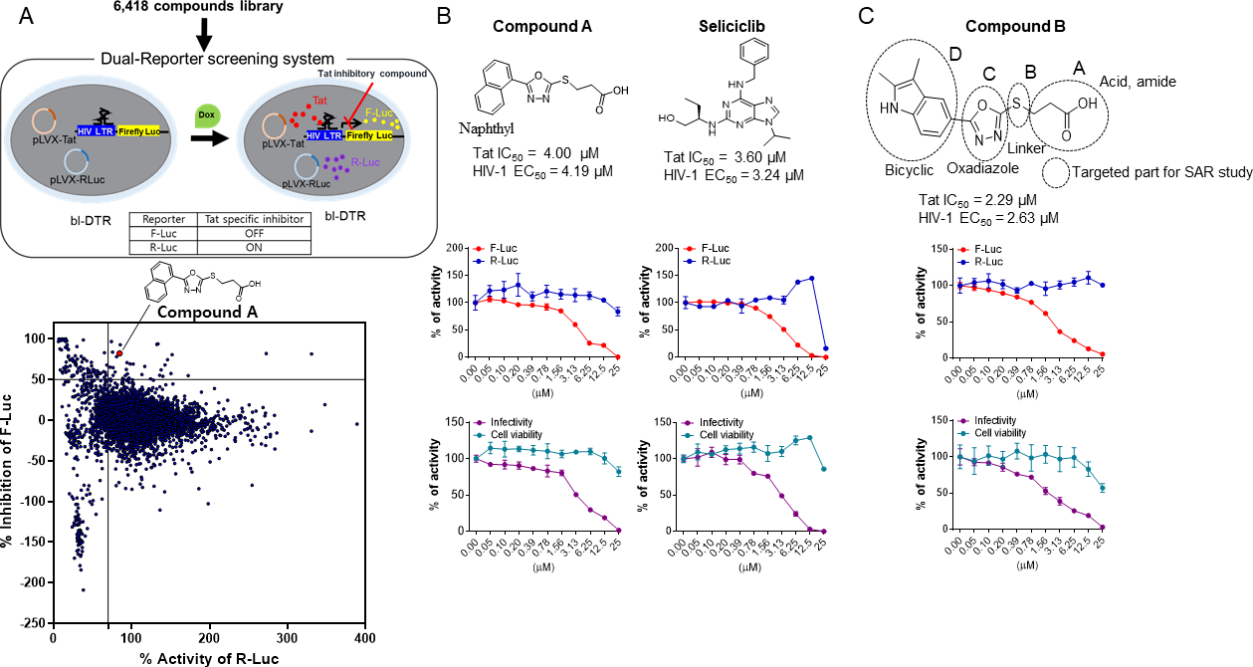
(**A**) (Top panel) Schematic representation of the primary compound screening using a dual-reporter system. (Bottom panel) Identification of primary hit compound A performed as described in Materials and Methods. (**B**). (Top panels) Chemical structures of the hit compound A and seliciclib (a Tat inhibitor). (Top graphs) bl-DTR cells (1 × 10^4^) were treated with twofold serial dilutions of each compound before doxycycline (Dox) addition (final concentration, 50 ng/mL). After 24 h of treatment, the activities of firefly luciferase (F-Luc, red line) and Renilla luciferase (R-Luc, blue line) were determined using the Dual-Glo Luciferase assay system. (Bottom graphs) TZM-bl cells were treated with each indicated compound and subsequently infected with the HIV-1_NL4-3_ strain at a multiplicity of infection (MOI) of 1. At 48 h after infection, the viral infectivity (purple line) and cell viability (green line) were determined using the Bright-Glo luciferase assay kit and the PrestoBlue Cell Viability Reagent, respectively. The relative activities are represented as the mean ± standard deviation (SD) (*n* = 3) compared with that of the dimethyl sulfoxide (DMSO) vehicle. (**C**). (Top panel) The chemical structure of compound B and its inhibitory effects on the Tat activity (top graph) and HIV-1 infectivity (bottom graph). The targeted moieties for the SAR study are marked with dotted-circle lines.

Our high through-put screen (HTS) of 6,418 compounds using a cellular assay based on dual reporters identified a hit compound **A** bearing a 1,3,4-oxadiazole core that has a potent inhibitory effect on firefly luciferase (F-Luc) activity (indicating Tat-mediated LTR transcription) but not on Renilla luciferase (R-Luc) activity (indicating general cellular transcription/translation) (Fig. 1A). The hit compound exhibited a half-maximal inhibitory concentration (IC_50_) value of 4.0 µM against Tat activity without a severe decrease of the R-Luc activity. Its effect was concordant with its inhibitory effect on HIV-1 infection without cytotoxicity. Compound **A** contained two structural moieties (1-naphthyl and carboxylic acid) connected to a 1,3,4-oxadiazole-2-thiol core. The potency of the hit compound **A** was similar to that of seliciclib (roscovitine or CYC202), a known Tat/HIV-1 inhibitor that disrupts cyclin-dependent kinase (CDK) activities, including those of CDK2-Cyclin E and CDK9-Cyclin T (22), exhibiting an IC_50_ of 3.60 µM against Tat activity (Fig. 1B). We enhanced the potency of the hit by firstly synthesizing a derivative (compound **B**) that transformed naphthyl in compound **A** to indole because indole is widely used for providing good drug properties in medicinal chemistry (23). Interestingly, the transformation demonstrated an increased inhibitory effect on Tat and HIV-1 infection (Fig. 1C). Given the enhanced potency, compound **B** was selected for a subsequent structure–activity relation (SAR) study to optimize the inhibitory effect on the biological activity.

### Chemical synthesis

**Fig 2:**
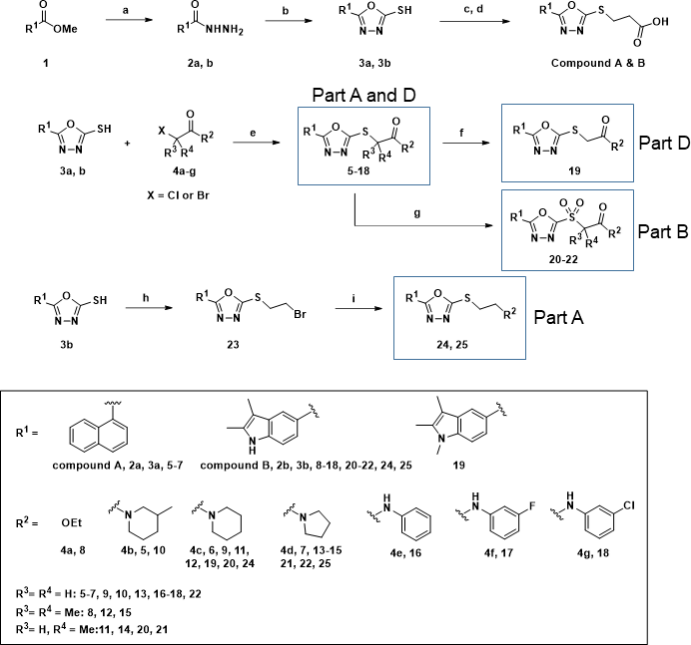
Synthesis of 5-aryl-1,3,4-oxadiazoles. Reagents and conditions: (a) NH_2_NH_2_·H_2_O, methanol, reflux, 12 h; (b) CS_2_, KOH, ethanol, reflux, 12 h; (c) *tert*-butyl 3-bromopropionate, KOH, MeOH, reflux, 6 h; (d) CF_3_CO_2_H, CH_2_Cl_2_, 25°C, 2 h; (e) K_2_CO_3_, ethanol, reflux, 8 h; (f) MeI, NaH, DMF, 25°C, 4 h; (g) *m*-chloroperoxybenzoic acid, CH_2_Cl_2_, 25°C, 8 h; (h) Br-CH_2_CH_2_-Br, K_2_CO_3_, acetone, 25°C, 4 h; (i) piperidine or pyrrolidine, K_2_CO_3_, DMF, 25°C, 4 h.

**Fig 3:**
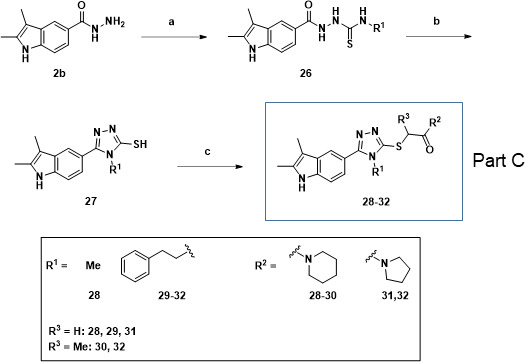
Synthesis of 5-indole-1,2,4-triazoles. Reagents and conditions: (a) alkyl isothiocyanates, triethylamine, ethanol, reflux, 3 h; (b) KOH, H_2_O, reflux, 10 h; (c) 2-chloro-acetamides, KOH, MeOH, 50°C, reflux, 3 h.

Compound **B** consisted of four parts: terminal carboxylic acid, thioalkyl linker, oxadiazole, and bicyclic parts. Considerable optimization was performed to study the SAR on compound **B**. We planned modification of all parts of the molecule (Fig. 1C). The synthesis of 5-aryl-1,3,4-oxadiazoles (compounds **A**, **B**, **5–22**, **24**, and **25**) was performed according to Fig. 2. Methyl esters **1** were treated with hydrazine hydrate in methanol (MeOH) under reflux to afford hydrazides **2a** and **2b,** which reacted with carbon disulfide in basic ethanol under reflux to give 1,3,4-oxadiazole-5-thiols **3a** and **3b**. Compounds **3a** and **3b** were alkylated with *tert*-butyl 3-bromopropionate in basic MeOH under reflux and subsequently hydrolyzed with trifluoroacetic acid in dichloromethane at 25°C to produce ([1,3,4-oxadiazol-2-yl]thio) propionic acids (compounds **A** and **B**). S-alkylation of **3a** and **3b** with 2-chloroacete **4a** or 2-halo-acetamides **4b-g** led to the final compounds **5–18**. Methylation of indolic NH of **13** with methyl iodide gave **19**. Oxidation of the selected sulfides (**11**, **13**, and **14**) with *m*-chloroperoxybenzoic acid (*m*-CPBA) afforded the final sulfones **20–22**. The 5-(2,3-dimethyl-1*H*-indol-5-yl)−1,3,4-oxadiazole-2-thiol (**3b**) was alkylated with 1,2-dibromoethane to produce alkyl halide **23**, which was directly alkylated with secondary amines to give final compounds **24** and **25** (Fig. 2). The synthesis of 5-aryl-1,2,4-triazoles (**28–32**) (derivatives of part C) was performed according to Fig. 3. The 2,3-Dimethyl-1*H*-indole-5-carbohydrazide **2b** reacted with alkyl isothiocyanates to afford thiosemicarbazides **26**, which were cyclized at reflux in aqueous potassium hydroxide to give corresponding 1,2,4-triazole-3-thiols **27**. The alkylation of **27** with 2-chloro-acetamides in basic MeOH led to the formation of final 1,2,4-triazole compounds **28–32** (Fig. 3).

### Testing the antiviral activity of analogs

**Fig 4:**
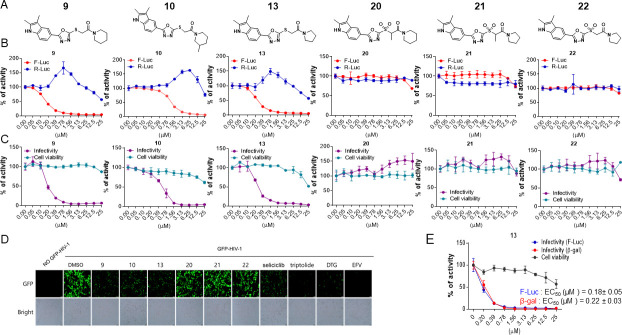
(**A**) Comparison of the chemical structures between prominent derivatives (9, 10, and 13) and non-functional derivatives (20, 21 and 22). (**B**) bl-DTR cells were treated with serial dilutions of each compound upon Dox for 24 h. The activities of F-Luc and R-Luc were determined using the Dual-Glo -Luciferase assay system. (**C**) TZM-bl cells were infected with the HIV-1_NL4-3_ strain at an MOI of 1 upon treatment of compounds. At 48 h after infection, the viral infectivity and cell viability were determined as described above (Fig. 1). (**D**) TZM-bl cells were infected with HIV-1_NL4-3_ IRES-eGFP virus along with the compound treatment. Two days later, GFP-expressing cells were observed by fluorescence microscopy (100× magnification). The treated concentrations were as follows: Compounds 9, 10, and 13 (1.5 μM); compounds 20, 21, and 22 (4 μM); seliciclib (10 µM); triptolide (5 nM); dolutegravir (DTG, 2 nM); efavirenz (EFV, 2 nM). (**E**) TZM-bl cells were infected with the HIV-1_NL4-3_ at an MOI of 1 upon compound 13. At 48 h after infection, the β-gal activity indicating viral infectivity was determined using the β-gal Enzyme Assay kit. F-Luc activity and cell viability were determined as described above (Fig. 4C). (**C and E**) The dose-response curves show the relative activity compared with that of the DMSO vehicle (mean ± SD; *n* = 3).

A series of analogs (**8–19**) were prepared from the precursor (**3b**) by appending the carbonyl analogs. Interestingly, in part A, the addition of the methylenecarbonyl group exhibited good inhibitory effects on Tat and HIV-1 activities without inhibitions of the R-Luc activity and cell viability (Fig. 2A through C). Compounds **9** and **13** showed half-maximal effective concentrations (EC_50_) of 0.17 and 0.24 µM, and 50% cytotoxicity concentrations (CC_50_) of 63.20 and 47.12 µM, respectively, resulting in corresponding selectivity indices (SIs) of 371.76 and 196.33 (Table 1). Triptolide and seliciclib as controls exhibited potent anti-HIV-1 activities with SI values of 52.63 and 12.10, respectively, similar to that shown in TZM-bl cells previously (**10, 12**) (Table 1). Adding methyl at the 3-position of the piperidine ring (**10**) decreased the anti-HIV-1 effect to approximately three-fold compared with the piperidine ring (**9**) (Fig. 4; Table 1). Interestingly, replacing anilines (**16–18**) at *R*^2^ increased Tat and HIV-1 activities without toxicity. Mono-methylation at α-carbon of carbonyl (**11** and **14**) decreased the potency against HIV-1 infection to approximately 2- to 13-fold compared with their un-methylated forms (**9** and **13**) (Table 1). Besides, di-methylation at the α-carbon of carbonyl (**12** and **15**) increased Tat and HIV-1 activities similarly to those shown by derivatives **16–18**. A series of derivatives (**20–22**) containing a sulfone at part B (Fig. 4A), which were derived from the effective derivatives (**9** and **13**), exhibited completely abrogated effects on the inhibitions of Tat and HIV-1 infection (Fig. 4B and C). A subsequent series of derivatives (**24** and **25**) modified in part A were prepared from a precursor (**3b**) by a sequential nucleophilic substitution reaction of 1,2-dibromoethane and piperidine or pyrrolidine (Fig. 2). The precursor (**3b**) did not exhibit a substantial biological effect on the inhibition of Tat or HIV-1 activity. A derivative (**24**) containing an S-ethyl-piperidine exhibited a modest inhibitory effect on Tat and HIV-1 infection with a 3.02 µM EC_50_ value against HIV-infection, but no effect was observed in the S-ethyl-pyrrolidine substituent (**25**) at the site (Table 1; ). We addressed the role of the oxadiazole core on the inhibitory effects of Tat and HIV-1 activities by synthesizing the triazole-modified derivatives (**28–32**) from a precursor (**2b**) (Fig. 3). The methyl-triazole one (**28**) exhibited a critical decrease of potency of the inhibitory activities against Tat and HIV-1. Meanwhile, the phenethyl-triazole modification (**29–32**) exhibited modest inhibitory effects on Tat and HIV-1 combined with a decrease in R-Luc activity (Table 2; Fig. S3). A subsequent SAR study focused on modifying the indole group (part D) of prominent derivatives (**9, 10,** and **13)** (Fig. S4). Derivatives **5–7** were synthesized under conditions similar to those used for synthesizing **8–18**, and derivative **19** was prepared by introducing a methyl group at the NH position of the indole in derivative **13** (Fig. 2). The naphthyl substituents (**5–7**) exhibited an approximately 50-fold lower inhibitory effect on Tat-mediated transcription than the prominent derivatives (**9, 10,** and **13**) but increased HIV-1 infectivity at some concentration ranges. The methyl-substituted indole compound **19** did not demonstrate any biological effect (Fig. S4). These data indicate that intact oxadiazole core and indole are crucial in inhibiting the Tat and HIV-1 activities. The cells were infected with enhanced green fluorescence protein (eGFP)-encoding HIV-1 (HIV-1_NL4-3_ IRES-eGFP-nef^+^) in the presence of each compound to confirm the potencies of prominent derivatives against HIV-1 infectivity with an orthogonal assay. Compounds (**9**, **10**, and **13**), two known Tat inhibitors (seliciclib and triptolide), and ARVs (dolutegravir, DTG and efavirenz, EFV) remarkably inhibited the viral-expressed eGFP signal, but the ineffective compounds (**20**, **21**, and **22**) did not (Fig. 4D; Fig. S2B). Compound **13** exhibited a dose-dependent inhibition of LTR-driven β-galactosidase (β-gal) activity in HIV-1 infected TZM-bl cells, and its inhibitory curve closely resembled that of the inhibition of F-Luc activity in the same cells (Fig. 4E). These data indicated that LTR-driven F-Luc activity from the TZM-bl cells may be closely associated with viral infectivity.

**TABLE 1:** Inhibitory effects of the derivatives substituted in part A on Tat and HIV-1^*e*^

Structure	Compound	IC_50_^*a*^ of F-Luc (Tat) (μM, mean ± SD)	Relative R-Luc activity at IC_50_ of F-Luc	EC_50_^*b*^ (μM, mean ± SD)	CC_50_^*c*^ (μM, mean ± SD)	SI^*d*^
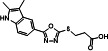	**B**	2.29 ± 0.08	>100	2.63 ± 0.34	44.63 ± 5.68	16.97
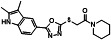	**9**	0.14 ± 0.00	>100	0.17 ± 0.02	63.20 ± 5.94	371.76
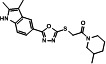	**10**	1.10 ± 0.07	>100	0.56 ± 0.05	82.47 ± 6.41	147.27
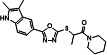	**11**	0.43 ± 0.03	100	0.40 ± 0.05	27.14 ± 1.95	67.85
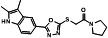	**13**	0.30 ± 0.03	>100	0.24 ± 0.03	47.12 ± 7.72	196.33
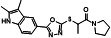	**14**	3.82 ± 0.12	>100	3.31 ± 0.28	57.74 ± 6.01	17.44
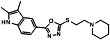	**24**	1.97 ± 0.03	>100	3.02 ± 0.09	40.28 ± 1.74	13.34
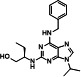	**Selic**	3.60 ± 0.10	>100	3.24 ± 0.07	39.21 ± 4.09	12.10
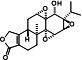	**Trip**	0.82 ± 0.02 nM	>100	0.38 ± 0.07 nM	0.02 ± 0.00	52.63

^*a*^ IC_50_: half-maximal inhibitory concentration; the IC_50_ values were determined in bl-DTR cells using the dose-response test upon doxycycline treatment.

^*b*^ EC_50_: half-maximal effective concentration.

^*c*^ CC_50_: 50% cytotoxicity concentration; the EC_50_ values were determined in TZM-bl cells infected with HIV-1_NL4-3_ at an MOI of 1 using the dose-response test. The CC_50_ values were determined in the absence of HIV-1 infection due to the viral cytopathic effect.

^*d*^ SI: selectivity index; SI values were calculated as CC_50_/EC_50_.

^*e*^ Undescribed derivatives (3b, 8, 12, 15, 16, 17, 18, and 25) exhibited no inhibitory effect on both Tat and HIV-1. Selic: seliciclib. Trip: triptolide.

**TABLE 2:** Inhibitory effects of the derivatives substituted in part C on Tat and HIV-1

Structure	Compound	IC_50_^*a*^ of F-Luc (Tat) (μM, mean ± SD)	Relative R-Luc activity at IC_50_ of F-Luc	EC_50_^*b*^ (μM, mean ± SD)	CC_50_^*c*^ (μM, mean ± SD)	SI^*d*^
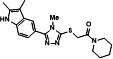	**28**	13.67 ± 0.53	90	21.15 ± 1.47	36.84 ± 12.61	1.74
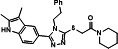	**29**	0.43 ± 0.03	90	0.39 ± 0.03	64.09 ± 6.31	164.33
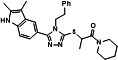	**30**	1.74 ± 0.06	100	2.01 ± 0.11	22.63 ± 0.47	11.26
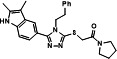	**31**	3.42 ± 0.12	>100	5.89 ± 0.05	55.32 ± 10.23	9.39
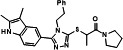	**32**	0.19 ± 0.01	90	0.17 ± 0.02	63.20 ± 2.65	371.76

^*a*^ IC_50_: half-maximal inhibitory concentration; the IC_50_ values were determined in bl-DTR cells using the dose-response test upon doxycycline treatment.

^*b*^ EC_50_: half-maximal effective concentration.

^*c*^ CC_50_: 50% cytotoxicity concentration; the EC_50_ values were determined in TZM-bl cells infected with HIV-1_NL4-3_ at an MOI of 1 using the dose-response test. The CC_50_ values were determined in the absence of HIV-1 infection due to the viral cytopathic effect.

^*d*^ SI: selectivity index; SI values were calculated as CC_50_/EC_50_.

### Determination of a step targeted by the compounds

**Fig 5:**
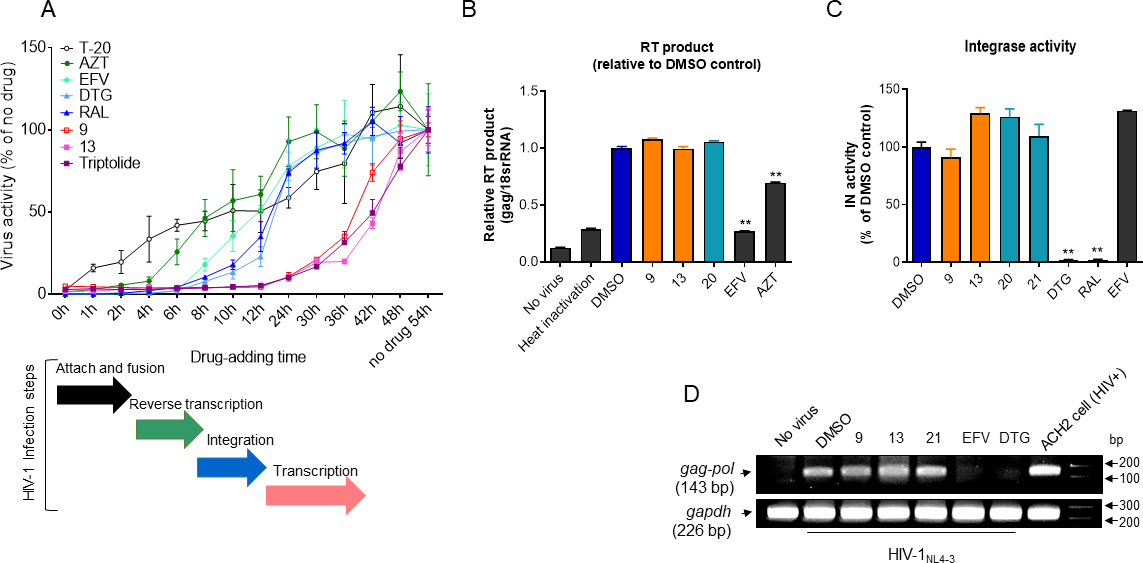
(**A**) Time-of-addition assay. TZM-bl cells were infected with HIV-1_NL4-3_ at an MOI of 1. Subsequently, the cells were treated with the compounds at the indicated time points (0, 1, 2, 4, 6, 8, 10, 12, 24, 30, 36, 42, and 48 h) after HIV-1 infection. The final concentrations of compounds are described in Materials and Methods. Viral activity according to the timing of drug addition was measured as F-Luc activity at 54 h after infection. The relative activities are represented as the mean ± SD (*n* = 3) compared with the activity observed 54 h post-infection. The relative time frames associated with each HIV-1 infection step are defined based on the time frame of susceptibility to the drug that blocks that particular infection step. (**B**) A3.01 cells (2 × 10^6^) were infected with DNase I-treated HIV-1_NL4-3_ at an MOI of 1 upon treatment of compounds (1.5 µM). At 16 h after infection, the viral reverse transcription (RT) product (viral cDNA) level was determined by quantitative real-time PCR (RT-qPCR) using *gag* and 18S rRNA primer sets and represented as the mean ± SD (*n* = 3) normalized against the amount of the 18S rRNA compared with the DMSO control. **P < 0.01 compared with the vehicle (DMSO). (**C**) An *in vitro* integrase (IN) assay was performed with 10 µM of the indicated compounds following the manufacturer’s protocol. **P < 0.01 compared with vehicle (DMSO). (**D**) A3.01 cells were infected with HIV-1 upon treatment with the indicated compounds (5 µM) and ARVs (EFV and DTG) as the experimental controls. At 40 h after infection, cellular chromosomal DNA was purified, and the integrated proviral DNA was detected through nested *Alu*-PCR amplification.

A time-of-addition assay was performed in parallel with well-defined ARVs as controls to address whether our prominent compounds specifically inhibited the viral transcription step. The HIV-1 virion attaches and fuses to the cell membrane, and the following steps serially occur: reverse transcription, integration, occasional cellular factors-dependent LTR-driven transcriptional initiation, and Tat-dependent viral transcriptional elongation (24). An LTR-downstream luciferase gene integrated into the chromosome of TZM-bl cells signifies that these processes occurred during HIV-1 infection. Therefore, this has been used widely as a readout for single-round viral infection (25, 26). The time-of-addition assay was calibrated using control ARV inhibiting distinct steps in the viral replication cycle (Fig. 5A). A drug becomes ineffective if it is administered after a targeted infection event. When the fusion inhibitor T-20 (or enfuvirtide) was administered 1 h after infection, its effectiveness began to decrease, consistent with a fusion event occurring soon after receptor binding. Moreover, the effectiveness of zidovudine (AZT) and EFV began to decrease when the compounds were administered 6 and 8 h after infection, respectively. This is consistent with AZT and EFV separately inhibiting the early and late stages of reverse transcription, as shown previously (25–27). The loss of effectiveness of the integrase inhibitors dolutegravir (DTG) and raltegravir (RAL) occurred when they were administered 10 h after infection. The effectiveness of triptolide, a known direct Tat inhibitor (10), disappeared upon its administration 24 h after infection, with data indicating that Tat-mediated viral transcription started at this time point under our experimental condition. Adding derivatives (**9** and **13**) blocked viral infectivity until 12 h after infection, while the loss of their activities suddenly occurred when they were administered 24 h after infection and was nearly complete 48 h after infection. The time kinetics of these compounds were closely similar to that exhibited by triptolide (Fig. 5A). Additionally, compounds **9** and **13** did not exhibit the inhibitory effect on the reverse transcription of the infected virus (Fig. 5B) and *in vitro* integrase activity (Fig. 5C). Furthermore, the nested *Alu*-PCR band for proviral DNA integrated to the host chromosome was not detected in cells infected with HIV-1 upon treatment with EFV (reverse-transcriptase inhibitor) or DTG (integrase inhibitor), but the proviral *gag-pol* band was observed upon treatment with compounds **9** or **13** as well as compound **21** (Fig. 5D). These results indicate that our compounds selectively inhibited Tat-mediated viral transcription apart from other viral infection steps.

### Inhibitory effect on the whole viral replication cycle

**Fig 6:**
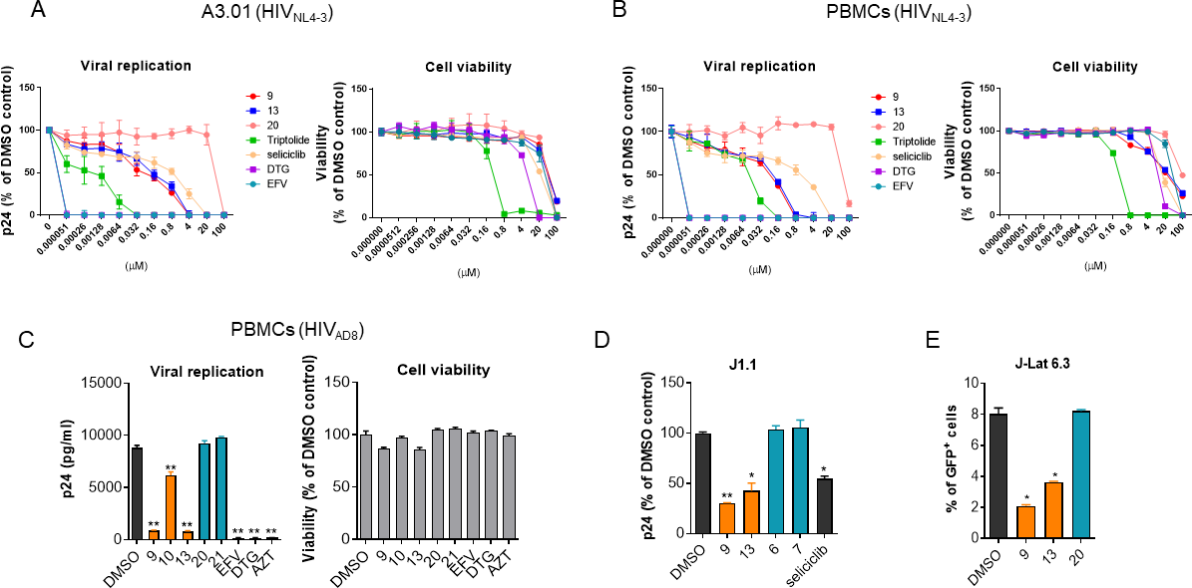
(**A**) A3.01 cells (5 × 10^4^) infected with HIV-1_NL4-3_ at an MOI of 0.1 were treated with fivefold serial dilutions of compounds or ARVs for 3 days. The p24 level from the cell supernatant was subsequently determined using a p24 ALPHALISA kit (PerkinElmer). In parallel, the cell viability was determined without viral infection because of the viral cytopathic effect. (**B**) Peripheral blood mononuclear cells (PBMCs) (4 × 10^5^) infected with HIV-1_NL4-3_ at an MOI of 0.1 were treated with serial dilutions of the compounds or ARVs for 3 days, and the p24 level was subsequently determined as above. In parallel, cell viability was determined. The data (**A and B**) are presented as the mean ± SD (*n* = 3). (**C**) PBMCs (4 × 10^5^) infected with HIV-_AD8_ at an MOI of 0.1 were treated with 1.5 µM of the compounds or ARVs for 3 days. The levels of p24 and cell viability were subsequently determined. (**D**) J1.1 cells were cultured with the compounds (3 µM) under 50 ng/mL phorbol myristate acetate (PMA) treatment for 2 days. The level of p24 was subsequently determined. (**E**) J-Lat 6.3 cells were treated with the compounds (1.5 µM) under 1 µg/mL of PMA for 2 days. The GFP-positive cell population was analyzed using flow cytometry. The data are represented as a value relative to the DMSO-treated vehicle (**D**) and a percent of the GFP^+^ cell population (**E**) as mean ± SD (*n* = 3). C-E. *P < 0.05 and **P < 0.01 compared with the cells treated with the vehicle (DMSO).

A3.01 T cells were infected with HIV-1_NL4-3_ and subsequently treated with fivefold serial dilutions of each compound for 3 days to evaluate the potency of our derivatives on the HIV-1 replication cycles. These prominent compounds (**9** and **13**) inhibited the viral replication, but the ineffective derivatives (20) did not (Fig. 6A). The EC_50_ values of compounds **9** and **13** were 0.25 and 0.51 µM, respectively, and their corresponding SI values were 224.40 and 93.29 (Table 3). Peripheral blood mononuclear cells (PBMCs) pre-activated with phytohemagglutinin M (PHA-M) were infected with CXCR4 tropic HIV-1_NL4-3_ upon treatment of each compound to evaluate their inhibitory effect on the overall HIV-1 life cycle in a primary cellular model. Compounds **9** and **13** effectively inhibited viral replication in PBMCs, and their corresponding SI values were 315.14 and 282.81 (Fig. 6B; Table 3). The potencies of our compounds were less than those of ARVs (DTG and EFV), but comparable with known Tat inhibitors (Fig. 4A and B; Table 3). The antiviral effect of our compounds was exerted on replication of CCR5 tropic HIV-1_AD8_ in PBMCs at 1.5 µM (approximate EC_90_ of compound **9** in TZM-bl cells) (Fig. 6C). In subsequent experiments with provirus reactivation, our compounds inhibited the viral production from the J1.1 cell line persistently infected with HIV-1 upon robust phorbol 12-myristate 13-acetate (PMA)-induced reactivation (Fig. 6D). The inhibitory effect on proviral reactivation was consistently observed in the J-Lat 6.3 cell line harboring a GFP-encoding HIV provirus (Fig. 6E). These data are consistent with inhibitory effects of these compounds on Tat-mediated HIV-1 transcription and HIV-1 infectivity.

**TABLE 3:** Inhibitory effects of the derivatives on HIV-1 replication

Compound	Cells	EC_50_^*a*^ (μM, mean ± SD)	CC_50_^*b*^ (μM, mean ± SD)	SI^*c*^
**9**	A3.01	0.25 ± 0.06	56.10 ± 4.48	224.40
	PBMCs	0.14 ± 0.01	44.12 ± 3.71	315.14
**13**	A3.01	0.51 ± 0.21	47.58 ± 1.88	93.29
	PBMCs	0.16 ± 0.02	45.25 ± 5.69	282.81
**seliciclib**	A3.01	3.59 ± 0.69	26.98 ± 1.12	7.52
	PBMCs	3.93 ± 0.30	21.44 ± 2.10	5.46
**triptolide**	A3.01	4.07 ± 2.30 nM	0.22 ± 0.04	54.05
	PBMCs	16.92 ± 3.75 nM	0.19 ± 0.00	11.23
**DTG**	A3.01	<1 nM	7.67 ± 0.61	>7,670
	PBMCs	<1 nM	18.27 ± 0.58	>18,270
**EFV**	A3.01	<1 nM	22.74 ± 1.65	>22,740
	PBMCs	<1 nM	29.46 ± 3.62	>29,460

^*a*^ EC_50_: half-maximal effective concentration.

^*b*^ CC_50_: 50% cytotoxicity concentration; The CC_50_ values were determined in the absence of HIV-1 infection due to the viral cytopathic effect.

^*c*^ SI: selectivity index; SI values were calculated as CC_50_/EC_50_.

### Inhibitory effect on ARV-resistant HIV-1 strains

**Fig 7:**
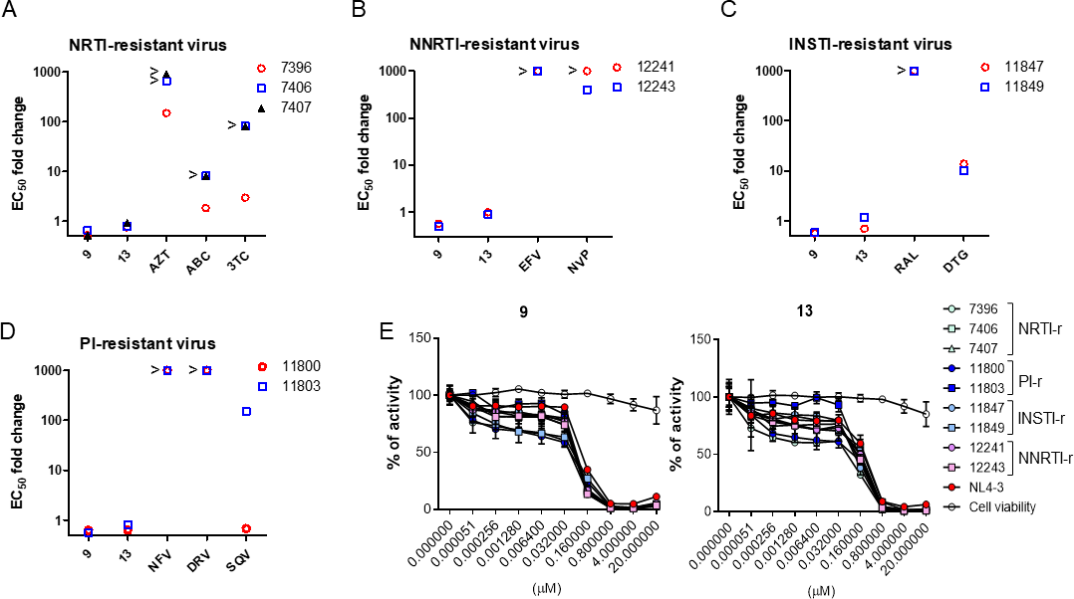
(**A–C**) TZM-bl cells (1 × 10^4^ cells/well) cultured in a 96-well plate were treated with fivefold serial dilutions of the indicated compound or ARV for 1 h before infection with the HIV-1_NL4-3_ or ARV-resistant HIV-1 strains at an MOI of 1, respectively. At 48 h after infection, the EC_50_ values were determined using an F-Luc activity. The symbols represent the fold-change values of EC_50_ against each mutant virus relative to the wild-type (WT) virus. (**D**) PBMCs (5 × 10^4^ cells/well) were infected with the HIV-1_NL4-3_ and PIs-resistant HIV-1 strain at an MOI of 0.1, respectively. Subsequently, the PBMCs were treated with 10-fold serial dilutions of the indicated compounds or PI. At 3 days after infection, the EC_50_ values on the viral replication were determined using a p24 ALPHALISA assay kit. (**E**) The inhibitory kinetics for the WT and ARV-resistant viruses. TZM-bl cells infected with WT or ARV-resistant viruses were cultured upon fivefold serial dilutions of each compound for 2 days and the infectivity was determined using an F-Luc activity. The data are presented as the relative activity compared with the DMSO vesicle (mean ± SD) (*n* = 3).

As our oxadiazole derivatives selectively inhibited a step of the Tat-dependent HIV-1 transcription, we attempted to determine whether these prominent compounds effectively inhibited the ARV-resistant mutant HIV-1 strains. Three nucleoside reverse-transcriptase inhibitor (NRTI)-resistant HIV-1 strains exhibited several 100-fold resistant EC_50_ values to AZT compared with wild-type HIV-1 as well as a mild resistance to abacavir (ABC) and lamivudine (3TC), except a 7396 strain treated with ABC. However, compounds **9** and **13** exhibited no resistance to these NRTI-resistant viruses (fold change of EC_50_: 0.52–0.93) (Fig. 7A; Table S1). Two non-nucleoside reverse-transcriptase inhibitors (NNRTI)-resistant HIV-1 strains exhibited great resistance to EFV and nevirapine (NVP), whereas the resistance was not observed for our compounds (Fig. 7B). Two integrase strand transfer inhibitor (INSTI)-resistant viruses were greatly resistant to RAL, but were moderately resistant to DTG (fold change of EC_50_: 9.92–13.75); however, both viruses were susceptible to our compounds similarly to that shown in the wild type (fold change of EC_50_: 0.56–1.16) (Fig. 7C; Table S1). The susceptibility of protease inhibitor (PI)-resistant viruses to our compounds was determined in PBMCs and TZM-bl cells infected with the resistant viruses. Two viruses that showed different resistances against the three PI types did not exhibit any resistance to our compounds (Fig. 7D). Dose-dependent inhibitory curves of the ARV-resistant HIV-1 strains upon administration of compounds **9** and **13** exhibited a parallel pattern to that of the wild-type virus (Fig. 7E). Moreover, EC_50_ values and dose-dependent inhibitory curves of each drug for ARV-resistant HIV-1 strains were comparable to those for wild-type HIV-1 (Table S1; Fig. S5).

### Molecular mechanism underlying the Tat inhibition of compounds

**Fig 8:**
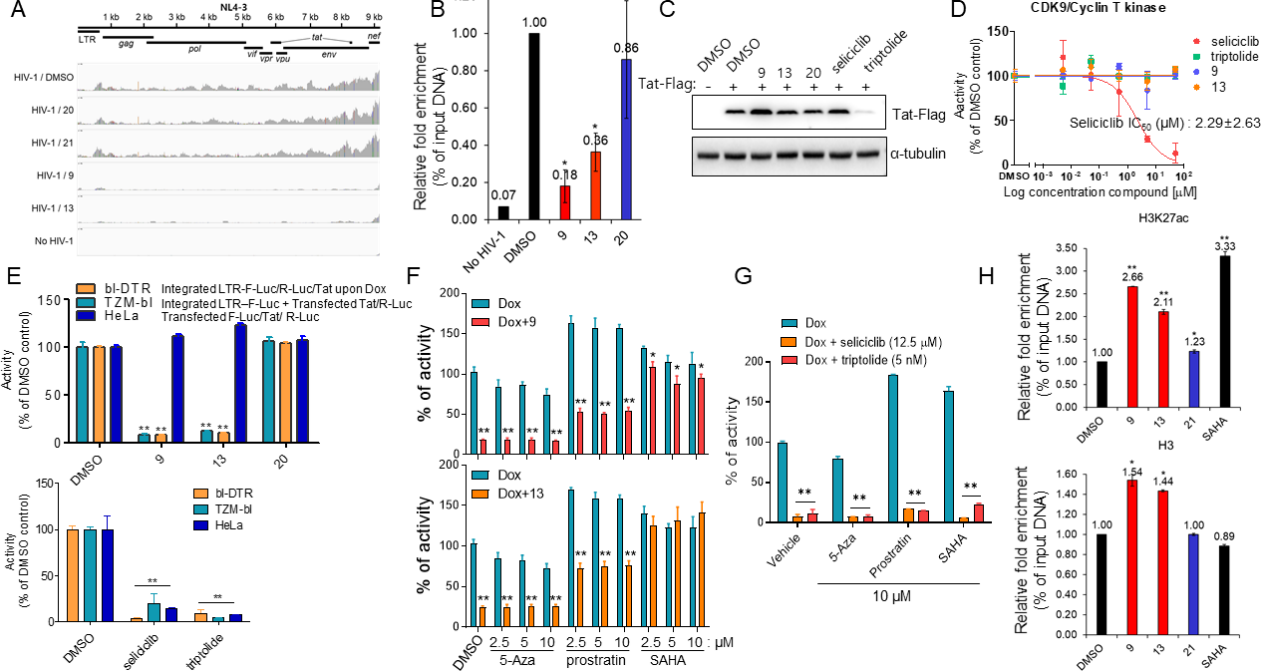
(**A**) TZM-bl cells infected with HIV-1 _NL4-3_ at an MOI of 1 were treated with the compounds (1.5 µM) for 2 days. The cDNA library was constructed and subsequently sequenced. The sequencing reads were subsequently aligned to the HIV-1 genome. The resulting BAM file, which represents the alignment to HIV-1_NL4-3_, was visualized using the Integrative Genomics Viewer (IGV) (29). (**B**) A3.01 cells (2 × 10^6^) infected with HIV-1 _NL4-3_ were treated with the compounds for 2 days. The cells were crosslinked, lysed, and immunoprecipitated with an anti-RNA pol II antibody. Immuno-complexed-chromatin was subjected to RT-qPCR using the *env* primer set. The data are expressed as the relative fold value of the percentage of the input compared with the vehicle (DMSO). (**C**) HeLa cells were transfected with pcDNA3-Flag-Tat86 plasmid. After 1 day, cells were treated with compounds (9, 13, or 20) (1.5 µM), seliciclib (12.5 µM), or triptolide (5 nM) for 2 days. The levels of Tat protein were analyzed by western blotting using antibodies against Flag and α-tubulin. (**D**) *In vitro* kinase activity was determined by serial dilutions of the compounds using the CDK9/Cyclin T Kinase Assay kit (BPS Bioscience) following the manufacturer’s instructions. The data are presented as the relative activity compared with the DMSO vesicle (mean ± SD) (*n* = 2). (**E**) Each cell line was transfected or treated with appropriate combinations as indicated following treatment with compounds (9, 13, or 20) (1.5 µM) (upper panel) or known Tat inhibitors [seliciclib (12.5 uM), triptolide (5 nM)] (bottom panel). Two days after treatment, Tat-induced LTR promoter activity was determined with F-Luc normalized to R-Luc activity. The data are represented as a relative percent of the vehicle (DMSO). (**F and G**) bl-DTR cells were treated with increasing amounts (0, 2.5, 5, and 10 µM) of epigenetic modulators following Dox exposure in the presence of the indicated compound (9 or 13) (1.5 µM) (**F**), known Tat inhibitors [seliciclib (12.5 µM) or triptolide (5 nM)] (**G**) Tat-induced LTR activity was measured as described above, 24 h after treatment. The data are represented as a relative percent of the vehicle (DMSO). (**H**) bl-DTR cells expressed Tat (Dox^+^) under the compounds (1.5 µM) for 24 h and were subsequently immunoprecipitated with the antibodies against histone H3 or H3K27ac. Immuno-complexed-chromatin was subjected to RT-qPCR using the Nuc-1 primer set. The data are expressed as a relative fold value of the percentage of the input compared with the vehicle (DMSO). (**B and E–H**) *P < 0.05 and **P < 0.01, compared with the cells treated with vehicle (DMSO).

To assess the inhibitory effect of our lead compounds on Tat-mediated HIV-1 transcription, RNA-seq and quantitative real-time PCR (RT-qPCR) analyses of the HIV-1 transcripts were performed in the compound-treated HIV-1-infected cells. The read counts of the transcripts mapped to the HIV-1 genome were substantially reduced in the compound **9**- or **13**-treated cells; however, the decrease of the read counts was not observed in the HIV-1 infected cells with the ineffective compounds (**20** and **21**) (Fig. 8A). Similar repression of the transcripts was observed in the RT-qPCR analysis (Fig. S6A). RNA pol II levels on the HIV-1 genomic region (*env*) were also reduced under these lead compounds (Fig. 8B). These data reinforce that the viral transcriptional elongation step is a target for our lead compounds. Subsequently, several experiments were conducted to gain insight into the possible molecular mechanism underlying Tat inhibition by these compounds. Compounds (**9** and **13**) did not exhibit a decrease of expression of Tat protein shown in triptolide (Fig. 8C), and an inhibition of CDK9/Cyclin T kinase activity shown in seliciclib (Fig. 8D). Moreover, these compounds did not affect the activity of the NF-κB-responsive element (RE), which is crucial for initiating HIV-1 transcription, under PMA or prostratin (Fig. S6B) and interaction between Tat and TAR RNA determined using a time-resolved fluorescence resonance energy transfer (TR-FRET) assay (Fig. S6C) (28). Subsequently, no changes were observed in the nuclear localization of the Tat protein (Fig. S6D) and in the interaction between Tat and cyclin T1 (Fig. S6E) in the compound (**9** and **13**)-treated cells. These data might indicate that these compounds indirectly inhibit the Tat function.

To address the Tat inhibitory pathway, Tat was expressed with chromosomal integrated or unintegrated LTR promoter-driven F-Luc under these lead compounds. The compounds abrogated the Tat activities [transfection- and doxycycline (Dox)-induced] in LTR-integrated cells (TZM-bl and bl-DTR); however, the inhibitory effect was not observed in the cells transfected with pLTR-F-Luc plasmid (Fig. 8E, upper panel). The phenomenon was not observed in treatment with known Tat inhibitors (seliciclib and triptolide) (Fig. 8E, bottom panel). These data indicate that our compounds target certain mechanisms related to Tat-associated epigenetic modulation of chromosome-integrated LTR. Cells were treated with epigenetic regulators during Tat inhibition by the compounds to elucidate the modes of action of the lead compounds. Treatment with 5-aza-2′-deoxycytidine (a DNA methyl transferase inhibitor) did not affect Tat activity (Dox-induced) inhibition by the compounds (**9** and **13**). Contrastingly, SAHA [vorinostate; a histone deacetylase (HDAC) inhibitor] and prostratin [a protein kinase C (PKC) activator] treatment further induced the activation of Tat-dependent LTR transcription. Otherwise, prostratin treatment did not affect the relative inhibitory effects of our compounds on Tat-dependent LTR transcription. In contrast, SAHA treatment nearly abrogated the inhibitory effects of our compounds on Tat activity (Fig. 8F). However, the abrogation effect by SAHA was not observed following treatment with the two known Tat inhibitors (seliciclib and triptolide) (Fig. 8G). In a chromatin immunoprecipitation (ChIP) assay for histone H3 and acetylated H3 at K27 (H3K27ac) on the LTR promoter, SAHA markedly increased the H3K27ac levels on the LTR promoter/nuc-1, whereas it decreased the total level of H3 on there, indicating histone ejection for facilitating RNA pol II elongation consistent with previous reports (30). Unlike SAHA, our compounds clearly increased both the levels of H3K27ac and H3 in nuc-1, indicating that the nucleosome ejection for RNA pol II elongation on LTR promoter might be interrupted by these compounds despite the high H3K27ac level on the LTR (Fig. 8H). Thus, the inhibition of Tat-mediated LTR transcription by our lead compounds may be associated with the inhibition of Tat-linked histone(s) (or protein[s]) acetylation on the LTR promoter.

## DISCUSSION

In this study, the HTS identified a 1,3,4-oxadiazole compound satisfying the inhibitory effect on Tat-mediated transcription corresponding to HIV-1 infection inhibition in a dose-dependent manner with low cellular toxicity. Oxadiazole-based compounds were widely synthesized in medicinal chemistry to develop new therapeutic agents due to their attractable biological activity. Among them, few oxadiazole derivatives have been demonstrated to have effective anti-HIV-1 activities. An oxadiazole derivative appended with other two types of rings and a naphthalene-oxadiazole hybrid bearing xylotetritolyl moiety exhibited potency against HIV-1 infection via allosteric inhibition of reverse-transcriptase with IC_50_ values of 8.5 and 1.44 µM, respectively (19, 20). Besides, incorporating oxadiazole into alkenyldiarylmethane (ADAMs) exhibited enhanced metabolic stability and the ability to inhibit HIV-1 reverse transcription (RT) for ADAMs (31). A series of indinavir derivatives bearing an oxadiazole demonstrated more potent inhibitory effects on the viral protease than indinavir (18). Concerning INSTI, an oxadiazole moiety on the RAL is known to participate in inhibiting the HIV-1 integrase strand transfer activity. Moreover, an oxadiazole substitution on naphthyridines exerted the greatest potency against HIV-1 integrase strand transfer activity (17). Despite the privileged scaffold of the oxadiazole nucleus with respect to different modes of action against HIV-1, their potential activities against Tat-mediated viral transcription have not yet been elucidated.

Here, we first discovered a hit compound (**A**) structured with a 1,3,4 oxadiazole core as an inhibitor of Tat-mediated viral transcription, following HIV-1 infection inhibition. Since an indole moiety is potent in multiple biological activities, including anti-HIV-1 activity (23), and is frequently observed in Tat inhibitory compounds, including guanidineindole, 6BIO, and aristolactams (12, 32, 33), we attempted to substitute a naphthyl moiety (in compound **A**) to indole (compound **B**). The substitution exerted a 1.7-fold improved inhibitory effect against Tat activity; therefore, a subsequent SAR study was conducted based on compound **B**. A piperidine ring substitution (24) lacking a ketone at part A exerted similar activity against Tat and HIV-1 compared with that shown by compound **B**, whereas the inhibitory effect disappeared with a pyrrolidine (25) (). Indeed, greater activities were exhibited by the piperidine amides than by the pyrrolidine amides with the same substituents in other parts (**9** vs **13** in Fig. 2, **11** vs **14** in Fig. S1, **6** vs **7** in Fig. S4). Considering that the basicities of the two rings are mostly similar, the bigger size of the piperidine ring is likely to be comparatively more suitable for binding to pocket(s) and/or on the interface(s) of certain molecule(s) for inhibiting Tat activity. Contrastingly, the arylamide group in part A (16–18) and dimethyl substitution at α-carbonyl in part B (**12** and **15**) increased the Tat and HIV-1 activities. Slight modifications leading to opposite actions suggest that certain key factor(s) may turn on or off the Tat-mediated transcriptional activity through allosteric regulation by our compounds. These compounds, which enhance the Tat and viral activities, could be potential candidates for developing a latency reversal agent in a “Kick and Kill” strategy (34, 35). The activity against Tat and HIV-1 entirely disappeared upon oxidation of sulfide to sulfone in part B (20–22) (Fig. 4). This suggests that the highly reactive sulfone may induce undesired chemical reactions, leading to instability and/or disruption of its inhibitory effect on Tat. Substitution of oxdiazole to triazole (28) critically abolished the potency of compound **9** against Tat and HIV-1. Contrastingly, the connection with phenethyl (30) demonstrated enhanced anti-Tat/HIV-1 activity compared with compound **28** (Fig. S3), similar to that observed with seliciclib (36, 37) and certain purine derivatives (38). However, it displayed non-specific inhibition of cellular transcription, as demonstrated by R-Luc activity. This suggests that the aryl moiety influences various cellular transcriptional regulators, similar to the inhibition of extracellular receptor-activated kinases (ERKs) observed with seliciclib (39) (Fig. S3).

Our promising compounds inhibited the viral transcription step in the time-of-addition assay (Fig. 5A) and demonstrated inhibition of the entire replication cycle in the T cell lines and PBMCs infected with HIV-1_NL-4-3_ and _-AD8_ (Fig. 4A through C). Furthermore, their inhibitory effects on provirus reactivation in latently infected cells were elucidated upon PMA treatment. However, the inhibitory effects were slightly less pronounced compared to their inhibitory effects on viral replication. This discrepancy is likely due to PMA-induced robust transcriptional initiation and elongation, independent of Tat activity (9, 40) (Fig. 6D and E). In a serial experiment, our compounds exhibited no influence on the interactions of Tat-TAR RNA and Tat-Cyc T1 and nuclear localization of Tat (Fig. S6C through E). Taken together, these data suggest that our compounds do not directly target the Tat, and their potency relies on a well-organized mechanism under healthy cellular conditions. Prostratin treatment did not abolish the inhibition of Tat-mediated LTR activity by our compounds. Moreover, the ~1.5-fold LTR activity increase induced by adding prostratin was sustained even in the presence of our compounds (Fig. 8F). Otherwise, our compounds did not affect the NF-κB RE activity by prostratin and PMA (Fig. S6B). Given that prostratin and PMA are established activators of PKC, which initiate transcriptional activation of the HIV-1 LTR through NF-κB activation following recruitment of histone acetyl transferases (HATs) such as p300/CBP for histone acetylation on LTR (41–44), these findings suggest that the inhibitory effect of our compounds on Tat-dependent transcription is likely distinct from that of the PKC-induced NF-κB pathway. Although SAHA treatment (which might enhance the histones and/or Tat acetylation levels) completely abrogated the Tat inhibitory effect of our compounds (Fig. 8F), HDAC activity in cellular nuclear extracts treated with our compounds remained unchanged even at high doses (10 and 100 µM) of the compounds (data not shown). This suggests the Tat inhibitory effect of these compounds might be linked to the acetylation status of protein(s) targeted by HDACs rather than the direct regulation of its deacetylase activity. Previous studies reported Tat interactions with HATs (P/CAF, p300/CBP, or GCN5) and its acetylations at Lys (K)−28 (by P/CAF) and at K50/K51 (by p300/CBP) along with the activities of Tat-associated HATs required for Tat-mediated LTR activation (45–48). Additionally, HDAC6 de-acetylated the Tat acetylation at K28 (49), resulting in Tat activity inhibition. Contrastingly, K50 de-acetylation by sirtuin1 was required for the complete Tat activity (50). Moreover, acetylated-Tat at K50 facilitated the recruitment of Switch/Sucrose-Nonfermentable (SWI/SNF) chromatin-remodeling complex (CRC) to the LTR promoter, resulting in remodeling of nuc-1 (40, 51). These findings together with ours, may support possible mechanisms underlying inhibition of Tat-mediated LTR activation by our compounds. The possible mechanisms include interference with Tat acetylation by HAT(s), disruption of the interaction between Tat and HATs (P/CAF or p300/CBP or GCN5), inhibition of Tat-associated HAT(s) activity for histone acetylation in the LTR, or prevention of the action of Tat-associated SWI/SNF remodeler on the LTR. The precise mode of action of our compounds against Tat activity remains to be elucidated and merits further investigation.

In conclusion, we identified novel oxadiazole compounds that inhibit HIV-1 Tat-mediated viral transcription. The SAR study involving serial synthesis of derivatives led to the identification of the most prominent compounds (**9** and **13**). These compounds exhibited potency against Tat transactivation activity toward HIV-1 replication without disruption of other viral processes. The compounds inhibited Tat-induced transcriptional elongation on the integrated HIV-1 promoter. Consequently, the inhibitory mechanism might be associated with inhibiting the Tat-mediated epigenetic regulation of the LTR promotor. Taken together, our results demonstrate that these potent compounds with an oxadiazole core could serve as a promising scaffold for developing novel therapeutic agents against drug-resistant HIV-1 infections.

## MATERIALS AND METHODS

### General chemistry

All reagents and solvents used in this study were purchased from commercial suppliers like Sigma-Aldrich, Alfa Aesar, TCI, Combi-blocks, or Angene and used without further purification. The organic solvents were evaporated using the Büchi rotary evaporator under reduced pressure and slightly heated temperature. The reaction progression was checked using thin-layer chromatography (Silica Gel 60F254 plate; Merck, Germany), and column chromatography was performed on the Merck silica gel (230–400 mesh) or in RediSep Rf normal-phase columns. Some compounds were purified using prep high-performance liquid chromatography (HPLC) with HPLC-grade solvents like acetonitrile, MeOH, and distilled water with 0.001% trifluoroacetic acid. Proton [^1^H nuclear magnetic resonance (NMR)] and carbon (^13^C) NMR spectra of the compounds were measured using Bruker Advance 300, 400, or 500 MHz spectrometer. The (CD_3_)_2_CO (2.05 ppm), CDCl_3_ (7.24 ppm), and (CD_3_)_2_SO (2.49 ppm) were used as the NMR solvents that were purchased from Cambridge, Inc. (MA, USA). Chemical shifts are provided in parts per million (ppm, δ) from downfield from tetramethylsilane (internal standard) with coupling constants in hertz (Hz). Multiplicity is indicated by the following abbreviations: singlet (s), doublet (d), triplet (t), and multiplet (m). The mass spectra of the compounds were determined using Agilent LC/MS, and high-resolution mass spectra were obtained from the Korea Research Institute of Chemical Technology using the fast-atom bombardment ionization method.

### Synthesis of the compounds

Detailed information about the synthesis of the compounds is described in the supplementary information.

### Cells, viruses, and reagents

The Tzm-bl-derived dual Tat reporter; TZM-bl/*Tat/Rluc* (bl-DTR) cells were established from TZM-bl cells using two Dox-inducible lentiviral expression cassettes cloned with Flag-tagged Tat86 and the R-Luc gene, as described in our previous study (11). TZM-bl, A3.01, J1.1, and J-Lat 6.3 cells as well as the HIV-1 molecular clone pNL4-3, pNL(AD8), ARV-resistant HIV-1 strains, and HIV-1_NL43_-IRES-eGFP (pBR43IeG-nef^+^, gifted by Dr. Frank Kirchhoff) (52) were provided by the National Institute of Health HIV Reagent Program (NIH, Bethesda, MD, USA). HeLa (CCL-2) and 293T (CRL-3216) cell lines were purchased from the American Type Culture Collection (ATCC, Manassas, VA, USA). The bl-DTR, TZM-bl, HeLa, and 293T cells were cultured in Dulbecco’s modified Eagle’s medium supplemented with 1% penicillin–streptomycin and 10% (vol/vol) heat-inactivated fetal bovine serum (all from Gibco-BRL, Gaithersburg, MD, USA), and 1 µg/mL of puromycin and 200 µg/mL of zeocin were additionally supplemented for the bl-DTR cells. PBMCs were purchased from AllCells (Alameda, CA, USA) and cultured as previously described (12). The information on ARV-resistant HIV-1 strains was described in previous reports as follows: NNRTI-resistant 12241 and 12243 (53); NRTI-resistant 7396, 7406, and 7407 (54); INSTI-resistant 11847 and 11849 (55, 56); and PI-resistant 11800 and 11803 (57). Each molecular HIV-1 clone was transfected to 293T cells, as previously described (58), to obtain infectious HIV-1 particles. Virus-containing cell culture supernatants were collected 3 days after transfection, and filtered through a 0.45-µm membrane filter (Millipore Sigma, Burlington, MA, USA) to remove cell debris. The viral titration was performed as described previously (59) with some minor modifications. In brief, 1 × 10^4^ TZM-bl cells seeded in 96-well plates were infected with 200 µL of virus-containing medium diluted serially twofold. The incubation was for 2 days and the experiment was performed in triplicate; the infection time minimized the virus spread (59). The titration of the infectious viral particles was performed with a ß-Gal staining assay using the ßeta-Galactosidase Staining Kit (Mirus Bio, Madison, WI, USA) following the manufacturer’s protocols. In brief, the infected cells were washed with phosphate-buffered saline (PBS) and fixed using a Cell Fixative Working solution. The cells were washed with PBS and stained using the Cell Staining Working Solution containing X-gal. The infected blue-colored cells were counted under a microscope (100× magnification). The infectious virus titer was determined as a mean value of blue-colored cells count × dilution factor and used for determining the multiplicity of infection (MOI) in each infection experiment. Seliciclib, AZT, ABC, 3TC, EFV, NVP, RAL, DTG, nelfiravir (NFV), darunavir (DRV), sequinavir (SQV), and triptolide were purchased from Sigma-Aldrich (St. Louis, MO, USA).

### Identification of primary hit compound

A library of 6,418 compounds was provided by the Korea Chemical Bank (www.chembank.org) of the Korea Research Institute of Chemical Technology. The screening was performed as previously described (12) with some minor modifications. In brief, for the primary screening, 1 × 10^4^ bl-DTR cells were cultured in 96-well white plates for 24 h and were treated with the compounds at 5 µM under 50 ng/mL of Dox. At 24 h after treatment, F-Luc and R-Luc activities were measured using the Dual-Glo-Luciferase Assay System (Promega, Madison, WI, USA) according to the manufacturer’s instructions. With several confirmatory tests, an oxadiazole compound (compound **A**) was identified among the compounds shown above approximately 50% inhibition of F-Luc activity without a substantial decrease in R-Luc activity.

### Inhibitory effects on the HIV-1 infectivity

The TZM-bl cells (also referred to as JC53BL-13) expressing human CD4, CXCR4, and CCR5 carrying the F-Luc and lacZ genes downstream of the HIV-1 LTR promoter (60) were used to determine the inhibitory effect of the compounds on HIV-1 infection, as previously described (11, 12, 61). In brief, 1 × 10^4^ cells were cultured in 96-well plates for 24 h and subsequently treated with compounds at the indicated concentrations. Without replacement of the culture medium, the cells were subsequently infected with the HIV-1_NL4-3_ wild-type or ARV-resistant HIV-1 strains at an MOI of 1 to determine the exact inhibitory effect of compounds on a single-round infection. After 48 h, the inhibitory effect of the compounds was determined using a Bright Glo luciferase assay kit (Promega) or β-Galactosidase Enzyme Assay kit (Promega). The infectivity data were presented as a percentage relative to the dimethyl sulfoxide (DMSO) control (vehicle). Cell viability was determined using the resazurin-based PrestoBlue Cell Viability Reagent (Thermo Fisher Scientific, Waltham, MA, USA) following the manufacturer’s instructions. Specifically, 7 × 10^4^ TZM-bl cells cultured in 24-well plates were infected with HIV-1_NL4-3_-eGFP-nef^+^ in the presence of indicated compounds to detect the inhibitory effect on the infectivity of GFP-encoding HIV-1. Two days after infection, the cells were washed with PBS, and GFP-expressing cells were detected by fluorescent microscopy (IX83, Olympus, Tokyo, Japan) and flow cytometry (FACSLyric, Becton Dickinson, Franklin Lakes, NJ, USA).

### Inhibitory effects on HIV-1 replication and reactivation

The inhibitory effects of the compounds on viral replication were determined with some minor modifications, as previously described (12, 62). In brief, 5 × 10^4^ cells/well of A3.01 cells were infected with HIV-1_NL4-3_ at an MOI of 0.1 with spinoculation at 300 × *g* for 2 h at 25°C, and treated with indicated compounds. PBMCs (4 × 10^5^) pre-activated with PHA-M for 3 days were washed with fresh media to remove excess PHA-M and subsequently infected with HIV-1_NL4-3_ or HIV-1_AD8_ or PIs-resistant HIV-1 strains as A3.01 infection. After infection, the infectious media were replaced with fresh media supplemented with international 10 units (IU)/mL of IL-2 and the indicated compounds. At 72 h after infection, the inhibitory effect of the compounds on viral replication was determined by measuring the p24 amount, an HIV capsid protein, using an HIV-1 p24 ALPHALISA kit (PerkinElmer, Waltham, MA, USA). Cell viability was determined as described above. The inhibitory effects on provirus reversing in J1.1 and J-Lat 6.3 cells were determined as previously described (12, 41, 63) with some minor modifications. Briefly, 5 × 10^4^ persistently HIV-1-infected J1.1 or J-Lat 6.3 cells were cultured in either the presence or absence of the compounds at a final concentration of 1.5 µM and treated with PMA (50 ng/mL for J1.1 cells, 1 µg/mL for J-Lat 6.3 cells). Two days after treatment, the levels of viral production were determined using p24 ALPHALISA for J1.1 cells and flow cytometry analysis for GFP-expressing J-Lat 6.3 cells.

### Time-of-addition assay

A time-of-addition assay was performed as previously described (25) with minor modifications. Briefly, 1 × 10^4^ cells/well of the TZM-bl cells were infected with HIV-1_NL4-3_ at an MOI of 1. Reference drug and test compounds were subsequently added at the indicated time (0, 1, 2, 4, 6, 8, 10, 12, 24, 30, 36, 42, and 48 h after infection). Final concentrations were as follows: compounds **9** and **13** (1.5 μM), T-20 (1 µM), Triptolide (5 nM), and ARVs (AZT, EFV, DTG, RAL) (1 µM). The viral activity according to the timing of drug addition was measured using a Bright Glo luciferase assay kit (Promega). Virus activity curves present a % activity relative to the activity observed at 54 h after infection, and the data are presented as the mean ± standard deviation (SD) (*n* = 3). The relative time frame associated with each HIV-1 infection step was defined based on the sensitivity to each drug that blocks a particular infection step.

### Quantification of the viral transcripts

We determined the level of viral transcripts using an RNA-seq analysis by infecting 5 × 10^5^ cells/well of TZM-bl cells with HIV-1_NL4-3_ at an MOI of 1 in six-well plates in the presence of the test compounds (1.5 µM). At 48 h after infection, the total RNA was extracted using the Maxwell RSC simplyRNA Cells Kit (Promega) according to the manufacturer’s instructions. RNA integrity was evaluated as RNA integrity number >6 using the Agilent 2100 Bio-analyzer (Agilent Technologies, Santa Clara, CA, USA). The cDNA libraries were constructed using TruSeq Stranded mRNA Sample Preparation Kit (Illumina, San Diego, CA, USA) according to the manufacturer’s instructions. Subsequently, these cDNA libraries were sequenced on the Illumina sequencing platform (NovaSeq6000; Illumina) and 150 bp paired-end reads were generated. Trimmomatic (v.0.32) was used to eliminate low-quality reads and adapter sequences from the sequencing data (64). Subsequently, the filtered reads were aligned to the HIV-1 genome (accession: AF324493.2) using STAR (v.2.3.0) (65). RT-qPCR analyses were performed as previously described (66) with some minor modifications. A3.01 cells (5 × 10^4^ cells/well) were infected with HIV-1_NL4-3_ at an MOI of 1 in 96-well plates. After infection, the infected cells were treated with 1.5 µM of the compounds. Forty-eight hours after treatment, the viral mRNA was isolated, converted to cDNA, and quantified through RT-qPCR on a 7500 Real-Time PCR System (Applied Biosystems) using the Power SYBR Green PCR Master Mix (Applied Biosystems). The comparative cycle threshold (Ct) method was used to analyze relative changes (Δ) in gene expression. The ΔCt values were determined using a 7500 Real-Time PCR System Software (Applied Biosystems) normalizing with *gapdh*, and the gene expression was calculated using the ΔΔCt-method. The primers used in this assay are the following: *gag* forward, 5′-GGTCCAAAATGCGAACCCAG-3′, *gag* reverse, 5′-TCTTGCTTTATGGCCGGGTC-3′; *gapdh* forward, 5′-GAAGGTGAAGGTCGGAGTC-3′, *gapdh* reverse, 5′-GAAGATGGTGATGGGATTTC-3′.

### ChIP assay

The ChIP assay was performed as previously described (12) according to the manufacturer’s protocol (Simple Chip Enzymatic chromatin IP kit, Cell Signaling). The cells were crosslinked with 1% formaldehyde for 10 min at 37°C and washed using ice-cold phosphate-buffered saline-containing protease inhibitors. The pelleted cells were resolved with a sodium dodecyl sulfate (SDS) lysis buffer and subsequently sonicated on ice. The sonicated samples were precleared using protein A/G-agarose and control immunoglobulin G for 30 min at 4°C. The precleared samples were then immuno-precipitated with antibodies against pol II (Cell Signaling Technology), histone H3 (Upstate), and histone H3K27ac (Upstate), overnight at 4°C, after which they were pulled down with protein A-agarose. The IP samples were reverse crosslinked and then digested with proteinase K. The recovered DNA was purified using the nucleic acid purification column provided in the kit (Cell Signaling Technology). The primers used in this assay are the following: Nuc-1 forward, 5′-AGCTTTCTACAAGGGACTTTCCGC-3′, Nuc-1 reverse, 5′-CTTCAGAGCAGACCAGAGCCA-3′; *env* forward, 5′-TTCAGCTACCACCGCTTGAG-3′, *env* reverse, 5′-TATGGCTGTGGCATTGAGCA-3′. The relative quantification of the precipitated chromatin was determined based on the threshold cycle of the PCR (CT) normalized to the control using the percent input method. Percent input = 100 × 2^[CT(adjusted input) – CT (IP)].

### Western blotting and co-immunoprecipitation

The protein levels of the cell lysates were analyzed using western blotting as previously described (12). In brief, whole-cell lysates and samples fractionated in nuclear or cytoplasm were prepared with a 5× SDS sample buffer and subsequently subjected to SDS-polyacrylamide gel electrophoresis. Proteins were subsequently transferred onto polyvinylidene difluoride membranes (Millipore). The protein levels on the membrane were analyzed using primary antibodies [against Flag M2 (Sigma-Aldrich), cyclin T1 (Santa Cruz Biotechnology), β-actin (Sigma-Aldrich), α-tubulin (Sigma-Aldrich), and lamin B (Santa Cruz Biotechnology)], secondary antibodies conjugated with horseradish peroxidase (HRP), and enhanced chemiluminescence western blotting detection reagents (Millipore).

The co-immunoprecipitation assay was performed as previously described (12, 58). Briefly, 293T cells were transfected with Tat-expressing plasmids (pcDNA3–Flag–Tat86) using Lipofectamine 2000 (Invitrogen). After 48 h, the cells were lysed in an immunoprecipitation buffer [250 mM NaCl, 0.5% NP-40, 50 mM Tris-HCl (pH 7.5), 5 mM ethylenediaminetetraacetic acid, and protease/phosphatase inhibitor cocktail]. The cell lysates were subsequently incubated with an anti-Flag monoclonal antibody (M2; Sigma-Aldrich) at 4°C. After overnight incubation, the immune complex was then pulled down with protein A/G-agarose beads (Santa Cruz Biotechnology) and analyzed using western blotting.

### HIV-1 RT and integration activities

The activity of HIV-1 RT was determined as previously described with some minor modifications (66–68). In brief, HIV-1_NL4-3_ obtained from plasmid transfection was treated with DNase I (2.5 U/mL) for 2 h at 37°C to remove the plasmid DNA. Specifically, 2 × 10^6^ of the A3.01 cells were infected with DNase I-treated active or heat-inactivated HIV-1_NL4-3_ (at an MOI of 1) in the presence of the indicated compounds. Sixteen hours after infection, the cell cytosolic DNA was isolated under RNase A treatment, and the RT products (cytosolic viral cDNA) were quantified using RT-qPCR and the following primers: *gag* forward, 5′-GGTCCAAAATGCGAACCCAG-3′, *gag* reverse, 5′-TCTTGCTTTATGGCCGGGTC-3′; 18S rRNA forward, 5′-GTAACCCGTTGAACCCCATT-3′, 18S rRNA reverse, 5′-CCATCCAATCGGTAGTAGGG-3′. The relative levels of RT products were analyzed using the ΔΔCt-method, as described above. *In vitro* integrase activity was determined using an HIV-1 integrase assay kit (XpressBio, Frederick, MD, USA) as previously described (12). In brief, a 96-well plate coated with donor substrate DNA was blocked, and HIV-1 integrase was then added to the plate. Subsequently, the compounds and target substrate DNA were added to the plate and incubated for 30 min at 37°C. The plate was washed and subsequently incubated with horse radish peroxidase. Integrase activity was measured by the peroxidase activity with the substrate at an optical density of 450 nM. The experiment was performed in triplicates. Detection of integrated proviral DNA was performed as previously described (69) with some minor modifications. Briefly, A3.01 cells were infected with HIV-1_NL4-3_ at an MOI of 1 and treated with the indicated compounds for 40 h. Chromosomal DNA was purified using the G-Spin DNA extraction kit (Intron Bio Tech, Seongnam-si, Gyeonggi-do, Republic of Korea). The integrated proviral HIV-1 DNA was detected by nested *Alu*-PCR amplification using the following primers: first round PCR, *Alu* forward, 5′-CTCACGCCTGTAATCCCAGCA-3′, *pol* reverse, 5′-TGTATCATCTGCTCCTGTATC-3′; second round PCR, *gag* forward, 5′-CCCTCTCAGAAGCAGGAGCCGA-3′, *pol* reverse. *gapdh* was used as the control gene.

### CDK9/Cyclin T kinase assay

CDK9/Cyclin T kinase assay was performed using the CDK9/Cyclin T Kinase Assay kit (BPS Bioscience, San Diego, CA, USA) following the manufacturer’s instructions. In brief, 12.5 µL of the Master Mix (Kinase Assay Buffer 1 supplemented with ATP and CDK Substrate Peptide 2) was mixed with 2.5 µL of each test compound diluted serially. The mixture was then mixed with CDK9/Cyclin T Kinase (50 ng/10 µL diluted in 1× Kinase Assay Buffer 1) and incubated for 45 min. The kinase activity was measured using Kinase-Glo Max (Promega). A decreased level of luminescence relative to ATP concentration indicated kinase activity, which was expressed as the percent of DMSO control.

### Statistical analysis

All data are expressed as the mean ± SD (*n* = 3), and the data were compared using a Student’s *t* test with statistical significance set at *P < 0.05 and **P < 0.01. All statistical analyses were performed using Prism (v.5.0; GraphPad, San Diego, CA, USA).

## Data Availability

The sequencing data are available in the Gene Expression Omnibus (GEO) database under accession number GSE273184.

## SUPPLEMENTAL MATERIAL

Supplemental material aac.00643-24-s0001.docx Supplemental methods; Table S1.

Supplemental figures aac.00643-24-s0002.pdf Figures S1 to S6.

ASM does not own the copyrights to Supplemental Material that may be linked to, or accessed through, an article. The authors have granted ASM a non-exclusive, world-wide license to publish the Supplemental Material files. Please contact the corresponding author directly for reuse.
