# Supplementary material for: Discovery of new acetamide derivatives of 5-indole-1,3,4-oxadiazol-2-thiol as inhibitors of HIV-1 Tat-mediated viral transcription
Source: Antimicrob Agents Chemother. 2024 Sep 4;68(10):e00643-24. doi: 10.1128/aac.00643-24 (PMC11459959; doi:10.1128/aac.00643-24)
Supplement: Supplemental material — Supplemental methods; Table S1. [file aac.00643-24-s0001.docx]

**SUPPLEMENTARY MATERIALS AND METHODS**

**Synthesis of the compounds**

**2,3-Dimethyl-1*H*-indole-5-carbohydrazide (2b)**

Methyl 2,3-dimethyl-1*H*-indole-5-carboxylate (6.0 g, 29.5 mmol) and hydrazine hydrate (40 mL, 686.4 mmol) in MeOH (10 mL) was refluxed for 12 h. The solvent was evaporated and white precipitates were washed with diethyl ether to give 4.0 g (67%) of **2b**.

^1^H NMR (400 MHz, DMSO-d6) δ 10.89 (s, 1H), 9.53 (s, 1H), 7.95 (d, *J* = 2.3 Hz, 1H), 7.52 (dd, *J* = 8.5, 1.7 Hz, 1H), 7.22 (d, *J* = 8.2 Hz, 1H), 4.40 (s, 2H), 2.31 (s, 3H), 2.18 (s, 3H). ^13^C NMR (100 MHz, DMSO-d6) δ 167.52, 136.69, 132.66, 128.37, 123.26, 119.30, 117.06, 109.65, 106.13, 11.21, 8.26. HRMS(EI): calcd for C_11_H_13_N_3_O, 203.1059; found 203.1053.

**5-(Naphthalen-1-yl)-1,3,4-oxadiazole-2-thiol (3a)**

To a solution of 1-naphthohydrazide **2a** (3.0 g, 16.11 mmol) in ethanol (80 mL) KOH (1.8 g, 32.22 mmol) in H_2_O (10 mL) was added. The mixture was stirred at 25 ^o^C for 10 min, and carbon disulfide (2.4 mL, 40.28 mmol) was subsequently added. After refluxing for 12 h, ethanol was evaporated and the aqueous layer was acidified by adding 1N HCl at pH = 3. The precipitates were collected by filtration, washed with water, and dried to afford 3.0 g (80%) of **3a**. ^1^H NMR (400 MHz, DMSO-d6) δ 14.93 (s, 1H), 8.82 (d, *J* = 8.6 Hz, 1H), 8.21 (d, *J* = 8.3 Hz, 1H), 8.14 – 8.06 (m, 2H), 7.75 – 7.64 (m, 3H). ^13^C NMR (100 MHz, DMSO-d6) δ 176.92, 160.34, 133.37, 132.99, 129.04, 128.65, 128.53, 128.38, 126.90, 125.35, 124.85, 118.53. HRMS(EI): calcd for C_12_H_8_N_2_OS, 228.0357; found 228.0357.

**5-(2,3-Dimethyl-1*H*-indol-5-yl)-1,3,4-oxadiazole-2-thiol (3b)**

The procedure used for **3a** was followed using 2,3-dimethyl-1*H*-indole-5-carbohydrazide (**2b**) (2.58 g, 12.7 mmol) to obtain **3b** (2.81 g, 90%). ^1^H NMR (400 MHz, DMSO-d6) δ 14.52 (s, 1H), 11.17 (s, 1H), 7.90 (s, 1H), 7.52 (dd, *J* = 8.5, 1.8 Hz, 1H), 7.38 (d, *J* = 8.5 Hz, 1H), 2.33 (s, 3H), 2.20 (s, 3H). ^13^C NMR (100 MHz, DMSO-d6) δ 177.43, 162.86, 137.52, 134.34, 129.30, 118.33, 116.67, 112.61, 111.68, 107.00, 106.89, 11.63, 8.57. HRMS(EI): calcd for C_12_H_11_N_3_OS, 245.0623; found 245.0614.

**3-((5-(Naphthalen-1-yl)-1,3,4-oxadiazol-2-yl)thio)propanoic acid (compound A)**

A mixture of 1,3,4-oxadiazole-2-thiol **3a** (50 mg, 0.22 mmol) and KOH (12 mg, 0.22 mmol) in methanol (1 mL) was stirred at 25 ^o^C. To the mixture, *tert*-Butyl 3-bromopropionate (0.055 mL, 0.33 mmol) was added, which was subsequently refluxed for 6 h. After concentration, the residue was dissolved in EtOAc, washed with H_2_O, dried over Na_2_SO_4_, and concentrated *in* *vacuo* to obtain crude alkylate, which was used in the next step without further purification.

The residue was dissolved in dichloromethane (1 mL) and trifluoroacetic acid (0.1 mL) and stirred for 2 h. After evaporation, the residue was purified by preparative HPLC (Shimpack PREP-ODS, H_2_O:CH_3_CN:CH_3_OH = 40:30:30 to 1:49.5:49.5, flow rate = 12 mL/min, 40°C, λ = 254 nm, retention time: 20 min) to afford compound A (42 mg, 64%). ^1^H NMR (400 MHz, Acetone-d6) δ 9.21 (d, *J* = 8.5 Hz, 1H), 8.20 (dd, *J* = 13.7, 7.8 Hz, 2H), 8.06 (d, *J* = 8.1 Hz, 1H), 7.76 – 7.64 (m, 3H), 3.62 (t, *J* = 6.8 Hz, 2H), 3.02 (t, *J* = 6.8 Hz, 2H). ^13^C NMR (100 MHz, DMSO-d6) δ 172.65, 166.42, 164.64, 134.90, 133.40, 130.59, 129.73, 129.19, 128.90, 127.66, 126.82, 126.09, 121.06, 34.45, 28.33. HRMS(EI): calcd for C_15_H_12_N_2_O_3_S, 300.0569; found 300.0563.

**3-((5-(2,3-Dimethyl-1*H*-indol-5-yl)-1,3,4-oxadiazol-2-yl)thio)propanoic acid (compound B)**

The procedure used for compound A was followed using 5-(2,3-dimethyl-1*H*-indole-5-yl)-1,3,4-oxadiazole-2-thiol (**3b**) (76 mg, 0.31 mmol) to obtain compound B (10 mg, 10%). ^1^H NMR (500 MHz, DMSO-d6) δ 12.53 (s, 1H), 11.14 (s, 1H), 7.98 (d, *J* = 1.6 Hz, 1H), 7.61 (dd, *J* = 8.4, 1.7 Hz, 1H), 7.39 (d, *J* = 8.4 Hz, 1H), 3.44 (t, *J* = 6.8 Hz, 2H), 2.82 (t, *J* = 6.8 Hz, 2H), 2.34 (s, 3H), 2.21 (s, 3H). ^13^C NMR (125 MHz, DMSO-d6): δ 172.48, 166.90, 162.11, 136.92, 133.71, 128.91, 118.36, 116.40, 112.94, 111.12, 106.43, 33.95, 27.60, 11.19, 8.17. HRMS(EI): calcd for C_15_H_15_N_3_O_3_S, 317.0834; found 317.0837.

**General procedures for synthesizing alkylated-1,3,4-oxadiazoles (5–18)**

The appropriate 1,3,4-oxadiazole-2-thiol (**3a** and **3b**, 1 equiv), 2-chloroacetamide or 2-chloroacetate (**4a**–**g**, 1.5 equiv), and KOH (1.0 equiv) were stirred in methanol at 60^°^C and the reaction progress was monitored via TLC. After evaporation, the residue was purified using preparative HPLC (Shimpack PREP-ODS, H_2_O:CH_3_CN:CH_3_OH = 50:25:25 to 1:49.5:49.5, flow rate = 12 mL/min, 40^°^C, λ = 254 nm, retention time: 25 min) to afford compounds **5**–**18**.

**1-(3-Methylpiperidin-1-yl)-2-((5-(naphthalen-1-yl)-1,3,4-oxadiazol-2-yl)thio)ethan-1-one (5)**

To a mixture of 1,3,4-oxadiazole-2-thiol **3a** (100 mg, 0.44 mmol) and KOH (25 mg, 0.44 mmol) in MeOH (2 mL), 2-chloro-1-(3-methylpiperidin-1-yl)ethan-1-one (115 mg, 0.66 mmol) was added. The mixture was stirred at 60°C for 3 h. After evaporation, the residue was purified using preparative HPLC to obtain compound **5** (49 mg, 32%). ^1^H NMR (400 MHz, DMSO-d6) δ 9.02 (d, *J* = 8.5 Hz, 1H), 8.21 (t, *J* = 8.9 Hz, 2H), 8.10 (d, *J* = 8.1 Hz, 1H), 7.77 – 7.65 (m, 3H), 4.68 – 4.54 (m, 2H), 4.25 – 4.13 (m, 1H), 3.84 – 3.75 (m, 1H), 3.21 – 2.85 (m, 1H), 2.70 (dd, *J* = 31.7, 11.5 Hz, 1H), 1.65 (dddd, *J* = 54.8, 35.4, 10.6, 4.1 Hz, 3H), 1.48 – 1.25 (m, 1H), 1.19 – 1.09 (m, 1H), 0.88 (dd, *J* = 17.9, 6.5 Hz, 3H). ^13^C NMR (100 MHz, DMSO-d6) δ 165.13, 164.58, 134.38, 133.62, 129.96, 129.90, 129.52, 129.24, 127.83, 126.35, 126.27, 120.33, 53.59, 49.83, 46.92, 43.13, 37.90, 37.85, 33.18, 33.12, 32.08, 31.51, 26.05, 25.42, 19.74, 19.59. HRMS(EI): calcd for C_20_H_21_N_3_O_2_S, 367.1354; found 367.1359.

**2-((5-(Naphthalen-1-yl)-1,3,4-oxadiazol-2-yl)thio)-1-(piperidin-1-yl)ethan-1-one (6)**

The procedure used for **5** was followed using 1,3,4-oxadiazole-2-thiol **3a** (100 mg, 0.44 mmol) and 2-chloro-1-(piperidin-1-yl)ethan-1-one (106 mg, 0.66 mmol) to obtain **6** (100 mg, 65%). ^1^H NMR (500 MHz, DMSO-d6) δ 9.03 (d, *J* = 8.6 Hz, 1H), 8.26 – 8.17 (m, 2H), 8.10 (d, *J* = 8.1 Hz, 1H), 7.77 – 7.66 (m, 3H), 4.61 (s, 2H), 3.48 (q, *J* = 5.9 Hz, 4H), 1.63 – 1.55 (m, 4H), 1.46 (dq, *J* = 10.7, 5.0 Hz, 2H). ^13^C NMR (125 MHz, DMSO-d6): δ 164.85, 164.18, 163.63, 133.43, 132.66, 128.97, 128.56, 128.29, 126.88, 125.36, 119.38, 46.44, 42.64, 36.95, 25.80, 25.19, 23.74. HRMS(EI): calcd for C_19_H_19_N_3_O_2_S, 353.1198; found 353.1185.

**2-((5-(Naphthalen-1-yl)-1,3,4-oxadiazol-2-yl)thio)-1-(pyrrolidin-1-yl)ethan-1-one (7)**

The procedure used for **5** was followed using 1,3,4-oxadiazole-2-thiol **3a** (100 mg, 0.44 mmol) and 2-chloro-1-(pyrrolidin-1-yl)ethan-1-one (97 mg, 0.66 mmol) to obtain **7** (104 mg, 70%). ^1^H NMR (500 MHz, DMSO-d6) δ 9.05 – 8.98 (m, 1H), 8.24 – 8.16 (m, 2H), 8.12 – 8.07 (m, 1H), 7.76 – 7.66 (m, 3H), 4.48 (s, 2H), 3.56 (t, *J* = 6.8 Hz, 2H), 3.35 (t, *J* = 6.9 Hz, 2H), 1.96 – 1.90 (m, 2H), 1.85 – 1.78 (m, 2H). ^13^C NMR (125 MHz, DMSO-d6): δ 164.85, 164.11, 163.61, 133.42, 132.64, 128.96, 128.51, 128.26, 126.86, 125.35, 119.36, 45.98, 36.79, 25.63, 23.97. HRMS(EI): calcd for C_18_H_17_N_3_O_2_S, 339.1041; found 339.1043.

**Ethyl 2-((5-(2,3-dimethyl-1*H*-indol-5-yl)-1,3,4-oxadiazol-2-yl)thio)-2-methyl-propanoate (8)**

The procedure used for **5** was followed using 5-(2,3-dimethyl-1*H*-indol-5-yl)-1,3,4-oxadiazole-2-thiol **3b** (200 mg, 0.82 mmol) and ethyl 3-bromo-2,2-dimethylpropanoate (238 mg, 1.22 mmol) to obtain **8** (190 mg, 64%). ^1^H NMR (500 MHz, CDCl3) δ 8.16 (d, *J* = 1.6 Hz, 1H), 8.01 (s, 1H), 7.78 (dd, *J* = 8.4, 1.7 Hz, 1H), 7.33 (d, *J* = 8.4 Hz, 1H), 4.20 (q, *J* = 7.1 Hz, 2H), 2.39 (s, 3H), 2.26 (s, 3H), 1.75 (s, 6H), 1.21 (t, *J* = 7.1 Hz, 3H).

^13^C NMR (125 MHz, CDCl_3_) δ 172.79, 168.82, 160.24, 137.36, 132.79, 129.79, 120.01, 117.98, 114.56, 110.83, 108.63, 62.31, 53.70, 26.64, 14.41, 14.17, 11.83, 8.61. HRMS(EI): calcd for C_18_H_21_N_3_O_3_S, 359.1304; found 359.1312.

**2-((5-(2,3-Dimethyl-1*H*-indol-5-yl)-1,3,4-oxadiazol-2-yl)thio)-1-(piperidin-1-yl)ethan-1-one (9)**

The procedure used for **5** was followed using 5-(2,3-dimethyl-1*H*-indol-5-yl)-1,3,4-oxadiazole-2-thiol **3b** (100 mg, 0.41 mmol) and 2-chloro-1-(piperidin-1-yl)ethan-1-one (99 mg, 0.61 mmol) to obtain **9** (49 mg, 32%).

^1^H NMR (400 MHz, DMSO-d6) δ 11.14 (s, 1H), 7.98 (s, 1H), 7.62 (d, *J* = 8.4 Hz, 1H), 7.39 (d, *J* = 8.4 Hz, 1H), 4.52 (s, 2H), 3.46 (d, *J* = 5.5 Hz, 4H), 2.33 (s, 3H), 2.20 (s, 3H), 1.58 (s, 4H), 1.49 – 1.41 (m, 2H). ^13^C NMR (100 MHz, DMSO) δ 166.73, 164.25, 162.18, 136.94, 133.73, 128.92, 118.38, 116.40, 112.96, 111.13, 106.42, 46.45, 42.62, 36.86, 25.82, 25.21, 23.78, 11.19, 8.18. HRMS(EI): calcd for C_19_H_22_N_4_O_2_S, 370.1463; found 370.1461.

**2-((5-(2,3-Dimethyl-1*H*-indol-5-yl)-1,3,4-oxadiazol-2-yl)thio)-1-(3-methylpiperidin-1-yl)ethan-1-one (10)**

The procedure used for **5** was followed using 5-(2,3-dimethyl-1*H*-indol-5-yl)-1,3,4-oxadiazole-2-thiol **3b** (100 mg, 0.41 mmol) and 2-chloro-1-(3-methylpiperidin-1-yl)ethan-1-one (108 mg, 0.61 mmol) to obtain **10** (65 mg, 41%). ^1^H NMR (500 MHz, DMSO-d6) δ 11.15 (s, 1H), 7.98 (d, *J* = 1.6 Hz, 1H), 7.62 (dd, *J* = 8.4, 1.7 Hz, 1H), 7.39 (d, *J* = 8.4 Hz, 1H), 4.59 – 4.47 (m, 2H), 4.22 – 4.10 (m, 1H), 3.83 – 3.74 (m, 1H), 3.24 – 3.01 (m, 1H), 2.76 – 2.60 (m, 1H), 2.37 (dd, *J* = 12.7, 10.5 Hz, 1H), 2.33 (s, 3H), 2.21 (s, 3H), 1.76 (dd, *J* = 13.0, 3.8 Hz, 1H), 1.72 – 1.50 (m, 2H), 1.50 – 1.30 (m, 1H), 1.17 – 1.09 (m, 1H), 0.87 (dd, *J* = 21.3, 6.6 Hz, 3H). ^13^C NMR (125 MHz, CDCl_3_) δ 167.67, 164.63 (d, *J* = 4.5 Hz), 163.14 (d, *J* = 4.1 Hz), 137.17, 132.71, 129.62, 119.72, 117.59, 114.33, 110.71, 108.36, 53.73, 49.87, 46.93, 43.09, 37.66 (d, *J* = 12.1 Hz), 32.89 (d, *J* = 5.0 Hz), 31.92, 31.07 (d, *J* = 2.7 Hz), 25.92, 24.77, 19.00 (d, *J* = 18.1 Hz), 11.70, 8.54. HRMS(EI): calcd for C_20_H_24_N_4_O_2_S, 384.1620; found 384.1626.

**2-((5-(2,3-Dimethyl-1*H*-indol-5-yl)-1,3,4-oxadiazol-2-yl)thio)-1-(piperidin-1-yl)propan-1-one (11)**

The procedure used for **5** was followed using 5-(2,3-dimethyl-1*H*-indol-5-yl)-1,3,4-oxadiazole-2-thiol **3b** (100 mg, 0.41 mmol) and 2-bromo-1-(piperidin-1-yl)propan-1-one (134 mg, 0.61 mmol) to obtain **11** (98 mg, 62%). ^1^H NMR (400 MHz, DMSO-d6) δ 11.15 (s, 1H), 7.98 (d, *J* = 2.3 Hz, 1H), 7.63 (dd, *J* = 8.5, 1.8 Hz, 1H), 7.39 (d, *J* = 8.5 Hz, 1H), 4.96 (q, *J* = 6.9 Hz, 1H), 3.60 – 3.47 (m, 3H), 3.42 – 3.37 (m, 1H), 2.34 (s, 3H), 2.21 (s, 3H), 1.60 (d, *J* = 6.6 Hz, 6H), 1.46 (t, *J* = 5.7 Hz, 2H). ^13^C NMR (100 MHz, DMSO-d6) δ 167.81, 167.24, 161.18, 136.99, 133.77, 128.92, 118.43, 116.48, 112.94, 111.16, 106.43, 46.58, 44.32, 42.71, 26.09, 25.25, 23.85, 19.67, 11.20, 8.17. HRMS(EI): calcd for C_20_H_24_N_4_O_2_S, 384.1620; found 384.1629.

**2-((5-(2,3-Dimethyl-1*H*-indol-5-yl)-1,3,4-oxadiazol-2-yl)thio)-2-methyl-1-(piperidin-1-yl)propan-1-one (12)**

The procedure used for **5** was followed using 5-(2,3-dimethyl-1*H*-indol-5-yl)-1,3,4-oxadiazole-2-thiol **3b** (50 mg, 0.20 mmol) and 2-bromo-2-methyl-1-(piperidin-1-yl)-propan-1-one (73 mg, 0.31 mmol) to obtain **12** (49 mg, 64%). ^1^H NMR (300 MHz, DMSO-d6) δ 11.20 (s, 1H), 7.96 (d, *J* = 1.6 Hz, 1H), 7.62 (dd, *J* = 8.5, 1.7 Hz, 1H), 7.43 – 7.39 (m, 1H), 3.68 (s, 4H), 2.34 (s, 3H), 2.22 – 2.19 (m, 3H), 1.68 (s, 6H), 1.58 (d, *J* = 18.3 Hz, 6H). ^13^C NMR (126 MHz, DMSO-d6) δ 169.02, 168.42, 160.10, 137.59, 134.38, 129.41, 118.89, 117.14, 113.28, 111.72, 106.89, 54.62, 28.27, 26.07, 24.41, 11.68, 8.57. HRMS(EI): calcd for C_21_H_26_N_4_O_2_S, 398.1776; found 398.1770.

**2-((5-(2,3-Dimethyl-1*H*-indol-5-yl)-1,3,4-oxadiazol-2-yl)thio)-1-(pyrrolidin-1-yl)ethan-1-one (13)**

The procedure used for **5** was followed using 5-(2,3-dimethyl-1*H*-indol-5-yl)-1,3,4-oxadiazole-2-thiol **3b** (100 mg, 0.41 mmol) and 2-chloro-1-(piperidin-1-yl)ethan-1-one (90 mg, 0.61 mmol) to obtain **13** (57 mg, 39%). ^1^H NMR (400 MHz, DMSO-d6) δ 11.19 (s, 1H), 7.97 (s, 1H), 7.65 – 7.58 (m, 1H), 7.39 (d, *J* = 8.5 Hz, 1H), 4.39 (s, 2H), 3.54 (t, *J* = 6.8 Hz, 2H), 3.32 (d, *J* = 6.5 Hz, 2H), 2.33 (s, 3H), 2.20 (s, 3H), 1.91 (q, *J* = 6.9 Hz, 2H), 1.83 – 1.77 (m, 2H). ^13^C NMR (101 MHz, DMSO-d6) δ 166.73, 164.20, 162.15, 136.93, 133.74, 128.91, 118.33, 116.36, 112.91, 111.14, 106.39, 46.12, 45.95, 36.80, 25.65, 23.97, 11.19, 8.17.

HRMS(EI): calcd for C_18_H_20_N_4_O_2_S, 356.1307; found 356.1329.

**2-((5-(2,3-Dimethyl-1*H*-indol-5-yl)-1,3,4-oxadiazol-2-yl)thio)-1-(pyrrolidin-1-yl)propan-1-one (14)**

The procedure used for **5** was followed using 5-(2,3-dimethyl-1*H*-indol-5-yl)-1,3,4-oxadiazole-2-thiol **3b** (100 mg, 0.41 mmol) and 2-bromo-1-(pyrrolidin-1-yl)propan-1-one (126 mg, 0.61 mmol) to obtain **14** (94 mg, 39%). ^1^H NMR (500 MHz, DMSO-d6) δ 11.15 (s, 1H), 7.97 (d, *J* = 1.6 Hz, 1H), 7.62 – 7.60 (m, 1H), 7.39 (d, *J* = 8.4 Hz, 1H), 4.70 (d, *J* = 6.9 Hz, 1H), 3.68 (dt, *J* = 10.0, 6.8 Hz, 2H), 3.56 (dt, *J* = 10.0, 6.9 Hz, 2H), 2.34 (s, 3H), 2.21 (s, 3H), 1.94 – 1.90 (m, 2H), 1.83 – 1.79 (m, 2H), 1.61 (d, *J* = 6.8 Hz, 3H). ^13^C NMR (125 MHz, DMSO-d6): δ 167.68, 167.21, 161.11, 136.98, 133.78, 128.91, 118.39, 116.43, 112.89, 111.16, 106.42, 46.26, 46.02, 45.11, 25.67, 23.83, 18.82, 11.19, 8.16. HRMS(EI): calcd for C_19_H_22_N_4_O_2_S, 370.1463; found 370.1466.

**2-((5-(2,3-Dimethyl-1*H*-indol-5-yl)-1,3,4-oxadiazol-2-yl)thio)-1-(pyrrolidin-1-yl)propan-1-one (15)**

The procedure used for **5** was followed using 5-(2,3-dimethyl-1*H*-indol-5-yl)-1,3,4-oxadiazole-2-thiol **3b** (50 mg, 0.20 mmol) and 2-bromo-2-methyl-1-(1-pyrrolidinyl)-1-propanone (68 mg, 0.31 mmol) to obtain **11** (49 mg, 64%). ^1^H NMR (400 MHz, CDCl_3_) δ 8.44 (s, 1H), 8.02 (d, *J* = 1.6 Hz, 1H), 7.65 (dd, *J* = 8.5, 1.7 Hz, 1H), 7.22 (d, *J* = 8.4 Hz, 1H), 3.99 (s, 2H), 3.59 (s, 2H), 2.34 (s, 3H), 2.20 (d, *J* = 0.6 Hz, 3H), 2.03 (s, 2H), 1.87 (s, 2H), 1.80 (s, 6H). ^13^C NMR (125 MHz, CDCl_3_) δ 169.61, 169.12, 159.76, 137.34, 132.79, 129.56, 119.69, 117.72, 114.07, 110.66, 108.23, 54.64, 48.90, 48.64, 27.59, 27.44, 23.36, 11.68, 8.47.

HRMS(EI): calcd for C_20_H_24_N_4_O_2_S, 384.1620; found 384.1623.

**2-((5-(2,3-Dimethyl-1*H*-indol-5-yl)-1,3,4-oxadiazol-2-yl)thio)-1-(pyrrolidin-1-yl)propan-1-one (16)**

The procedure used for **5** was followed using 5-(2,3-dimethyl-1*H*-indol-5-yl)-1,3,4-oxadiazole-2-thiol **3b** (100 mg, 0.41 mmol) and chloroacetanilide (104 mg, 0.61 mmol) to obtain **16** (47 mg, 30%). ^1^H NMR (400 MHz, DMSO-d6) δ 11.14 (s, 1H), 10.45 (s, 1H), 7.96 (s, 1H), 7.60 (d, *J* = 8.1 Hz, 3H), 7.39 – 7.29 (m, 3H), 7.08 (t, *J* = 7.4 Hz, 1H), 4.33 (s, 2H), 2.32 (s, 3H), 2.16 (s, 3H). ^13^C NMR (125 MHz, DMSO-d6): δ 166.90, 165.06, 161.80, 136.91, 133.71, 128.87, 123.67, 119.16, 118.33, 116.38, 112.84, 111.11, 106.41, 36.79, 11.18, 8.11. HRMS(EI): calcd for C_20_H_18_N_4_O_2_S, 378.1150; found 378.1146.

**2-((5-(2,3-Dimethyl-1*H*-indol-5-yl)-1,3,4-oxadiazol-2-yl)thio)-*N*-(3-fluorophenyl)acetamide (17)**

The procedure used for **5** was followed using 5-(2,3-dimethyl-1*H*-indol-5-yl)-1,3,4-oxadiazole-2-thiol **3b** (100 mg, 0.41 mmol) and 2-chloro-*N*-(3-fluorophenyl)acetamide (115 mg, 0.61 mmol) to obtain **16** (65 mg, 40%). ^1^H NMR (500 MHz, DMSO-d6) δ 11.14 (s, 1H), 10.65 (s, 1H), 7.94 (d, *J* = 1.6 Hz, 1H), 7.59 (td, *J* = 8.6, 2.0 Hz, 2H), 7.38 – 7.34 (m, 2H), 7.32 (dt, *J* = 8.4, 1.4 Hz, 1H), 6.92 (td, *J* = 7.5, 1.9 Hz, 1H), 4.33 (s, 2H), 2.33 (s, 3H), 2.16 (s, 3H). ^13^C NMR (125 MHz, DMSO-d6): δ 166.92, 165.52, 161.70, 136.92, 133.73, 130.57 (d, *J* = 9.4 Hz), 128.88, 118.32, 116.36, 114.93, 112.82, 111.12, 110.17 (d, *J* = 20.4 Hz), 106.07, 105.86, 36.75, 11.18, 8.08. HRMS(EI): calcd for C_20_H_17_FN_4_O_2_S, 396.1056; found 396.1054.

***N*-(3-Chlorophenyl)-2-((5-(2,3-dimethyl-1*H*-indol-5-yl)-1,3,4-oxadiazol-2-yl)thio)acetamide (18)**

The procedure used for **5** was followed using 5-(2,3-dimethyl-1*H*-indol-5-yl)-1,3,4-oxadiazole-2-thiol **3b** (100 mg, 0.41 mmol) and 2-chloro-*N*-(3-chlorophenyl)acetamide (125 mg, 0.61 mmol) to obtain **18** (28 mg, 17%). ^1^H NMR (500 MHz, DMSO-d6) δ 11.14 (s, 1H), 10.64 (s, 1H), 7.94 (d, *J* = 1.7 Hz, 1H), 7.80 (t, *J* = 2.1 Hz, 1H), 7.59 (dd, *J* = 8.5, 1.7 Hz, 1H), 7.46 (ddd, *J* = 8.2, 2.0, 1.0 Hz, 1H), 7.38 – 7.34 (m, 2H), 7.15 (ddd, *J* = 8.0, 2.1, 0.9 Hz, 1H), 4.32 (s, 2H), 2.33 (s, 3H), 2.16 (s, 3H). ^13^C NMR (125 MHz, DMSO-d6): δ 166.92, 165.56, 161.69, 140.08, 136.92, 133.73, 133.18, 130.61, 128.89, 123.41, 118.62, 118.32, 117.57, 116.36, 112.81, 111.12, 106.40, 36.73, 11.18, 8.08. HRMS(EI): calcd for C_20_H_17_ClN_4_O_2_S, 412.0761; found 412.0759.

**1-(Pyrrolidin-1-yl)-2-((5-(1,2,3-trimethyl-1*H*-indol-5-yl)-1,3,4-oxadiazol-2-yl)thio)ethan-1-one (19)**

To a solution of compound **13** (50 mg, 0.14 mmol) in DMF (1 mL) NaH (60% in mineral oil) (10 mg, 0.25 mmol) was added at 0^o^C. After stirring for 10 min at 25 ^o^C, MeI (30 mg, 0.21 mmol) was added at 0^o^C. The reaction mixture was stirred for 4 h at room temperature and quenched with water. The residue was diluted with EtOAc, washed with H_2_O, dried over Na_2_SO_4_, and purified by silicagel flash chromatography (hexane:EtOAc = 1:1) to afford **19** (25 mg, 48%). ^1^H NMR (400 MHz, CDCl_3_) δ 8.33 (d, *J* = 1.6 Hz, 1H), 7.92 (dd, *J* = 8.6, 1.7 Hz, 1H), 7.22 (d, *J* = 8.6 Hz, 1H), 4.89 (s, 2H), 3.66 (s, 3H), 3.55 (t, *J* = 6.9 Hz, 2H), 3.51 (t, *J* = 6.8 Hz, 2H), 2.35 (s, 3H), 2.28 – 2.26 (m, 3H), 2.04 – 1.97 (m, 2H), 1.91 – 1.85 (m, 2H). ^13^C NMR (100 MHz, CDCl_3_) δ 167.74, 165.86, 139.39, 134.26, 128.15, 122.67, 121.63, 119.75, 108.24, 108.10, 62.49, 46.18, 45.68, 29.90, 26.38, 24.09, 10.35, 8.93.

**2-((5-(2,3-Dimethyl-1*H*-indol-5-yl)-1,3,4-oxadiazol-2-yl)sulfonyl)-1-(piperidin-1-yl)propan-1-one (20)**

To a mixture of sulfide **11** (112 mg, 0.28 mmol) in dichloromethane (3 mL) m-chloroperbenzoic acid (m-CPBA, max 77%) (69 mg, 0.62 mmol) was added at 0^o^C. After stirring at 25 ^o^C for 8 h, the reaction mixture was quenched with aqueous sodium sulfite and stirred for 10 min. The residue was diluted with dichloromethane, washed with H_2_O, dried over Na_2_SO_4_, and purified using silicagel flash chromatography (MeOH:dichloromethane = 1:20) to afford **20** (25 mg, 30%). ^1^H NMR (300 MHz, DMSO-d6) δ 11.29 (s, 1H), 8.43 (d, *J* = 6.6 Hz, 1H), 8.41 (s, 1H), 8.15 (dd, *J* = 8.7, 2.1 Hz, 1H), 5.02 (q, *J* = 6.8 Hz, 1H), 3.50 (dq, *J* = 14.3, 7.1 Hz, 4H), 2.69 (s, 3H), 2.16 (s, 3H), 1.63 (s, 3H), 1.61 – 1.38 (m, 6H). ^13^C NMR (125 MHz, DMSO-d6) δ 201.52, 169.13, 167.72, 164.52, 162.77, 141.26, 131.44, 129.11, 125.30, 121.40, 117.31, 46.58, 44.81, 42.69, 28.72, 26.05, 25.19, 24.72, 23.77, 19.67. HRMS(EI): calcd for C_20_H_24_N_4_O_4_S, 416.1518; found 416.1537.

**2-((5-(2,3-Dimethyl-1*H*-indol-5-yl)-1,3,4-oxadiazol-2-yl)sulfonyl)-1-(pyrrolidin-1-yl)propan-1-one (21)**

The procedure used for **20** was followed using sulfide **14** (104 mg, 0.28 mmol) to obtain **21** (32 mg, 28%). ^1^H NMR (400 MHz, CDCl_3_) δ 11.87 (s, 1H), 8.92 (d, *J* = 8.9 Hz, 1H), 8.54 (d, *J* = 2.1 Hz, 1H), 8.11 (dd, *J* = 8.9, 2.1 Hz, 1H), 4.82 – 4.77 (m, 1H), 3.85 – 3.79 (m, 1H), 3.57 – 3.49 (m, 3H), 2.05 – 2.01 (m, 2H), 1.91 (t, *J* = 6.6 Hz, 2H), 1.75 (d, *J* = 7.0 Hz, 3H).

^13^C NMR (100 MHz, DMSO-d6) δ 201.57, 169.17, 167.59, 164.55, 162.69, 141.30, 131.45, 129.14, 125.25, 121.40, 117.29, 46.29, 46.04, 45.49, 28.75, 25.67, 24.76, 23.83, 18.84. HRMS(EI): calcd for C_19_H_22_N_4_O_4_S, 402.1362; found 402.1346.

**2-((5-(2,3-Dimethyl-1*H*-indol-5-yl)-1,3,4-oxadiazol-2-yl)sulfonyl)-1-(pyrrolidin-1-yl)ethan-1-one (22)**

The procedure used for **20** was followed using sulfide **141** (100 mg, 0.28 mmol) to obtain **22** (34 mg, 31%). ^1^H NMR (400 MHz, DMSO-d6) δ 11.29 (s, 1H), 8.42 (d, *J* = 8.8 Hz, 1H), 8.38 (s, 1H), 8.11 (d, *J* = 8.8 Hz, 1H), 4.42 (s, 2H), 3.54 (t, *J* = 6.9 Hz, 2H), 3.32 (d, *J* = 7.0 Hz, 2H), 2.68 (s, 3H), 2.15 (s, 3H), 1.92 (t, *J* = 6.9 Hz, 2H), 1.83 – 1.78 (m, 2H). ^13^C NMR (100 MHz, DMSO-d6) δ 201.66, 169.19, 164.13, 164.07, 163.69, 141.29, 131.43, 129.10, 125.11, 121.34, 117.30, 46.16, 45.99, 36.97, 28.79, 25.67, 24.79, 23.99. HRMS(EI): calcd for C_18_H_20_N_4_O_4_S, 388.1205; found 388.1209.

**2-((2-Bromoethyl)thio)-5-(2,3-dimethyl-1*H*-indol-5-yl)-1,3,4-oxadiazole (23)**

To a solution of compound **3b** (200 mg, 0.82 mmol) in acetone (5 mL) K_2_CO_3_ (340 mg, 2.46 mmol) and 1,2-dibromoethane (0.14 mL, 1.62 mmol) were added. After stirring for 4 h at 25 ^o^C, the reaction mixture was filtered, diluted with EtOAc, and washed with water. The resulting solution was concentrated *in vacuo* and the residue was purified using flash column chromatography to give **23** (100 mg, 35 %). ^1^H NMR (400 MHz, DMSO-d6) δ 11.14 (s, 1H), 7.98 (s, 1H), 7.62 (d, *J* = 8.4 Hz, 1H), 7.39 (d, *J* = 8.4 Hz, 1H), 3.87 (t, *J* = 7.3 Hz, 2H), 3.75 (t, *J* = 7.4 Hz, 2H), 2.34 (s, 3H), 2.21 (s, 3H). ^13^C NMR (100 MHz, DMSO-d6) δ 167.06, 161.43, 136.97, 133.73, 133.69, 128.92, 118.39, 116.45, 113.00, 112.90, 111.13, 106.45, 33.95, 31.08, 11.20, 8.17. HRMS(EI): calcd for C_14_H_14_BrN_3_OS, 351.0041; found 351.0055.

**2-(2,3-Dimethyl-1*H*-indol-5-yl)-5-((2-(piperidin-1-yl)ethyl)thio)-1,3,4-oxadiazole (24)**

To a solution of compound **23** (32 mg, 0.09 mmol) in DMF (1 mL) K_2_CO_3_ (25 mg, 0.18 mmol) and piperidine (15 mg, 0.18 mmol) were added. After stirring for 2 h at 25 ^o^C, the residue was filtered. The resulting solution was concentrated in vacuo and purified by flash column chromatography to give **24** (17.8 mg, 56 %). ^1^H NMR (400 MHz, DMSO-d6) δ 11.13 (s, 1H), 7.97 (s, 1H), 7.61 (dd, *J* = 8.4, 1.7 Hz, 1H), 7.38 (d, *J* = 8.4 Hz, 1H), 3.44 (t, *J* = 6.8 Hz, 2H), 2.67 (t, *J* = 6.8 Hz, 2H), 2.39 (t, *J* = 5.3 Hz, 4H), 2.33 (s, 3H), 2.20 (s, 3H), 1.44 (p, *J* = 5.5 Hz, 4H), 1.34 (q, *J* = 5.5 Hz, 2H). ^13^C NMR (100 MHz, DMSO-d6) δ 166.64, 162.87, 136.90, 133.71, 128.93, 118.32, 116.32, 113.09, 111.15, 106.41, 57.29, 53.69, 30.31, 25.50, 23.94, 11.21, 8.18. HRMS(EI): calcd for C_19_H_24_N_4_OS, 356.1671; found 356.1696.

**2-(2,3-Dimethyl-1*H*-indol-5-yl)-5-((2-(pyrrolidin-1-yl)ethyl)thio)-1,3,4-oxadiazole (25)**

To a solution of compound **23** (32 mg, 0.09 mmol) in DMF (1 mL) K_2_CO_3_ (25 mg, 0.18 mmol) and piperidine (15 mg, 0.18 mmol) were added. After stirring for 2 h at 25 ^o^C, the residue was filtered. The resulting solution was concentrated in vacuo and purified by flash column chromatography to give **24** (17.8 mg, 56 %).

^1^H NMR (400 MHz, CDCl_3_) δ 8.35 (s, 1H), 8.08 (s, 1H), 7.77 – 7.70 (m, 1H), 7.30 (d, *J* = 8.4 Hz, 1H), 3.48 (t, *J* = 7.0 Hz, 2H), 2.98 (t, *J* = 7.0 Hz, 2H), 2.65 (d, *J* = 6.0 Hz, 4H), 2.38 (s, 3H), 2.24 (s, 3H), 1.86 – 1.79 (m, 4H). ^13^C NMR (101 MHz, CDCl_3_) δ 167.56, 163.16, 137.06, 132.70, 129.49, 119.48, 117.36, 114.26, 110.80, 110.66, 108.14, 77.27, 54.81, 53.89, 31.29, 29.71, 23.54, 11.60, 8.42. HRMS(EI): calcd for C_18_H_22_N_4_OS, 342.1514; found 342.1527.

**5-(2,3-Dimethyl-1*H*-indol-5-yl)-4-methyl-4*H*-1,2,4-triazole-3-thiol (27b)**

Following the same procedure used for the synthesis of **27a**, carbohydrazide **2b** (250 mg, 1.23 mmol), methyl isothiocyanate (0.09 mL, 1.36 mmol), and KOH (152 mg, 2.71 mmol) were used to obtain **27b** (90 mg, 28%).

^1^H NMR (400 MHz, DMSO-d6) δ 13.77 (s, 1H), 11.04 (s, 1H), 7.70 (d, *J* = 1.7 Hz, 1H), 7.37 (d, *J* = 8.3 Hz, 1H), 7.29 (dd, *J* = 8.3, 1.6 Hz, 1H), 3.55 (s, 3H), 2.34 (s, 3H), 2.19 (s, 3H). ^13^C NMR (100 MHz, DMSO-d6) δ 167.03, 153.16, 136.03, 133.29, 128.81, 120.24, 118.28, 115.68, 110.63, 106.05, 54.92, 31.74, 11.23, 8.22. HRMS(EI): calcd for C_13_H_14_N_4_S, 258.0939; found 258.0934.

**2-((5-(2,3-Dimethyl-1*H*-indol-5-yl)-4-methyl-4*H*-1,2,4-triazol-3-yl)thio)-1-(piperidin-1-yl)ethan-1-one (28)**

The procedure used for **5** was followed using **27b** (36 mg, 0.14 mmol) and 2-bromo-1-(piperidin-1-yl)ethan-1-one (34 mg, 0.21 mmol) to obtain **28** (38 mg, 71%).

^1^H NMR (400 MHz, Acetone-d6) δ 10.24 (s, 1H), 7.86 (s, 1H), 7.43 (q, *J* = 8.4 Hz, 2H), 4.41 (s, 2H), 3.84 (s, 3H), 3.54 (d, *J* = 5.6 Hz, 4H), 2.40 (s, 3H), 2.24 (s, 3H), 1.65 (s, 6H). ^13^C NMR (101 MHz, DMSO-d6) δ 164.70, 136.20, 133.53, 128.88, 120.37, 118.52, 114.38, 110.80, 106.12, 46.47, 42.52, 37.28, 32.26, 25.82, 25.18, 23.79, 11.22, 8.20. HRMS(EI): calcd for C_20_H_25_N_5_OS, 383.1780; found 383.1781.

**2-(2,3-Dimethyl-1*H*-indole-5-carbonyl)-*N*-phenethylhydrazine-1-carbothioamide (26a)**

To a solution of carbohydrazide **2b** (500 mg, 2.46 mmol) in EtOH (10 mL) phenethyl isothiocyanate (0.4 mL, 2.71 mmol) and triethylamine (0.72 mL, 5.17 mmol) were added. After refluxing for 8 h, the product was precipitated after cooling of the reaction mixture. The precipitated residue was filtered and washed with EtOH to give **26a** (600 mg, 66%). ^1^H NMR (400 MHz, DMSO-d6) δ 10.96 (s, 1H), 10.13 (s, 1H), 9.28 (s, 1H), 8.07 (s, 2H), 7.63 – 7.58 (m, 1H), 7.29 – 7.24 (m, 3H), 7.24 – 7.17 (m, 3H), 3.68 – 3.61 (m, 2H), 2.82 (t, *J* = 7.8 Hz, 2H), 2.32 (s, 3H), 2.20 (s, 3H). ^13^C NMR (100 MHz, DMSO-d6) δ 167.26, 139.40, 137.12, 132.93, 128.63, 128.41, 128.28, 126.10, 122.26, 120.17, 118.15, 109.66, 106.33, 45.33, 35.01, 11.26, 8.33. HRMS(EI): calcd for C_20_H_22_N_4_OS, 366.1514, found: 366.1533.

**5-(2,3-Dimethyl-1*H*-indol-5-yl)-4-phenethyl-4*H*-1,2,4-triazole-3-thiol (27a)**

To a mixture of compound **26a** (600 mg, 1.64 mmol) in water (10 mL) was added KOH (280 mg, 4.99 mmol) at 25 ^o^C. After stirring for 12 h at 90°C, the reaction mixture was cooled to room temperature and acidified with 1N HCl to pH 2. The precipitated product was filtered, washed with H_2_O, and dried under vacuum to give **27a** (428 mg, 75%).

^1^H NMR (400 MHz, DMSO-d6) δ 13.81 (s, 1H), 11.03 (s, 1H), 7.42 (s, 1H), 7.34 (d, *J* = 8.3 Hz, 1H), 7.20 (d, *J* = 6.6 Hz, 3H), 7.11 – 7.06 (m, 1H), 6.99 (dd, *J* = 7.1, 2.2 Hz, 2H), 4.21 (dd, *J* = 9.1, 6.3 Hz, 2H), 3.10 – 2.90 (m, 2H), 2.34 (s, 3H), 2.17 (s, 3H). ^13^C NMR (100 MHz, DMSO-d6) δ 166.46, 153.17, 137.49, 135.92, 133.24, 128.69, 128.52, 128.49, 126.53, 120.25, 118.21, 115.47, 110.56, 105.96, 45.17, 32.99, 11.22, 8.24. HRMS(EI): calcd for C_20_H_20_N_4_S, 348.1409; found 348.1414.

**5-(2,3-Dimethyl-1*H*-indol-5-yl)-4-phenethyl-4*H*-1,2,4-triazole-3-thiol (29)**

The procedure used for **5** was followed using **27a** (143 mg, 0.41 mmol) and 2-chloro-1-(piperidin-1-yl)ethan-1-one (79 mg, 0.49 mmol) to obtain **29** (167 mg, 86%).

^1^H NMR (400 MHz, DMSO-d6) δ 11.06 (s, 1H), 7.44 (s, 1H), 7.35 (d, *J* = 8.3 Hz, 1H), 7.20 (t, *J* = 3.4 Hz, 3H), 7.14 – 7.09 (m, 1H), 6.99 – 6.93 (m, 2H), 4.25 (t, *J* = 7.5 Hz, 2H), 4.21 (s, 2H), 3.42 (q, *J* = 5.9 Hz, 4H), 2.88 (t, *J* = 7.5 Hz, 2H), 2.34 (s, 3H), 2.18 (s, 3H), 1.56 (q, *J* = 8.2 Hz, 4H), 1.43 (p, *J* = 5.5 Hz, 2H). ^13^C NMR (100 MHz, DMSO-d6) δ 165.00, 156.72, 149.10, 137.10, 135.69, 133.05, 128.80, 128.61, 128.53, 126.70, 120.34, 117.88, 116.94, 110.57, 105.83, 46.49, 45.53, 42.48, 37.45, 34.96, 25.87, 25.19, 23.82, 11.25, 8.30. HRMS(EI): calcd for C_27_H_31_N_5_OS, 473.2249; found 473.2247.

**2-((5-(2,3-Dimethyl-1*H*-indol-5-yl)-4-phenethyl-4*H*-1,2,4-triazol-3-yl)thio)-1-(piperidin-1-yl)propan-1-one (30)**

The procedure used for **5** was followed using **27a** (143 mg, 0.41 mmol) and 2-bromo-1-(piperidin-1-yl)propan-1-one (108 mg, 0.49 mmol) to obtain **30** (167 mg, 79%). ^1^H NMR (400 MHz, DMSO-d6) δ 10.99 (s, 1H), 7.50 (s, 1H), 7.37 (d, *J* = 8.3 Hz, 1H), 7.21 – 7.19 (m, 2H), 7.19 – 7.13 (m, 2H), 6.93 (dd, *J* = 6.6, 2.7 Hz, 2H), 4.72 (t, *J* = 6.9 Hz, 1H), 4.24 (t, *J* = 7.4 Hz, 2H), 3.47 (d, *J* = 7.7 Hz, 4H), 2.82 (t, *J* = 7.3 Hz, 2H), 2.35 (s, 3H), 2.20 (s, 3H), 1.56 (q, *J* = 4.8 Hz, 3H), 1.51 (s, 3H), 1.47 (d, *J* = 6.8 Hz, 3H). ^13^C NMR (100 MHz, DMSO-d6) δ 168.43, 156.89, 148.05, 137.01, 135.70, 133.07, 128.83, 128.55, 128.52, 126.71, 120.33, 117.91, 117.03, 110.59, 105.89, 46.51, 45.48, 43.28, 42.61, 35.01, 26.12, 25.22, 23.88, 19.29, 11.25, 8.30. HRMS(EI): calcd for C_28_H_33_N_5_OS, 487.2406; found 487.2403.

**2-((5-(2,3-Dimethyl-1*H*-indol-5-yl)-4-phenethyl-4*H*-1,2,4-triazol-3-yl)thio)-1-(pyrrolidin-1-yl)ethan-1-one (31)**

The procedure used for **5** was followed using **27a** (143 mg, 0.41 mmol) and 2-chloro-1-(pyrrolidin-1-yl)ethan-1-one (72 mg, 0.49 mmol) to obtain **31** (139 mg, 74%). ^1^H NMR (400 MHz, Acetone-d6) δ 10.23 (s, 1H), 7.62 (s, 1H), 7.42 (d, *J* = 8.2 Hz, 1H), 7.21 (q, *J* = 4.5 Hz, 4H), 7.08 – 7.02 (m, 2H), 4.45 (t, *J* = 7.8 Hz, 2H), 4.25 (s, 2H), 3.60 (t, *J* = 6.7 Hz, 2H), 3.39 (d, *J* = 6.9 Hz, 2H), 3.05 (t, *J* = 7.6 Hz, 2H), 2.40 (s, 3H), 2.24 (s, 3H), 2.01 – 1.97 (m, 2H), 1.86 (t, *J* = 7.0 Hz, 2H). ^13^C NMR (100 MHz, DMSO-d6) δ 164.82, 136.98, 135.79, 133.15, 128.78, 128.59, 128.52, 126.72, 120.31, 118.03, 116.07, 110.60, 105.90, 46.12, 45.88, 45.67, 37.72, 34.78, 25.65, 23.94, 11.22, 8.26. HRMS(EI): calcd for C_26_H_29_N_5_OS, 459.2093; found 459.2108.

**2-((5-(2,3-Dimethyl-1*H*-indol-5-yl)-4-phenethyl-4*H*-1,2,4-triazol-3-yl)thio)-1-(pyrrolidin-1-yl)propan-1-one (32)**

The procedure used for **5** was followed using **27a** (143 mg, 0.41 mmol) and 2-bromo-1-(pyrrolidin-1-yl)propan-1-one (108 mg, 0.49 mmol) to obtain **32** (151 mg, 78%). ^1^H NMR (400 MHz, DMSO-d6) δ 11.05 (s, 1H), 7.53 – 7.50 (m, 1H), 7.38 (d, *J* = 8.3 Hz, 1H), 7.21 – 7.19 (m, 2H), 7.17 (dd, *J* = 8.4, 1.7 Hz, 2H), 6.93 (dd, *J* = 6.8, 2.7 Hz, 2H), 4.47 (t, *J* = 6.8 Hz, 1H), 4.26 (q, *J* = 7.6 Hz, 2H), 3.51 (d, *J* = 6.7 Hz, 2H), 3.29 (d, *J* = 6.9 Hz, 2H), 2.84 (t, *J* = 7.4 Hz, 2H), 2.35 (s, 3H), 2.19 (s, 3H), 1.88 – 1.83 (m, 2H), 1.75 (dt, *J* = 7.0, 5.2 Hz, 2H), 1.49 (d, *J* = 6.7 Hz, 3H). ^13^C NMR (101 MHz, DMSO-d6) δ 168.12, 136.85, 135.93, 133.32, 128.83, 128.55, 128.53, 126.75, 120.24, 118.11, 115.69, 110.73, 105.99, 46.19, 45.94, 45.70, 44.72, 34.84, 25.62, 23.81, 18.45, 11.23, 8.26. HRMS(EI): calcd for C_27_H_31_N_5_OS, 473.2249; found 473.2242.

**SUPPLEMENTARY TABLE**

**Supplementary Table S1. HIV-1 resistance to ARVs and compounds 9/13.**

| Type of HIV-1 | Virus | Drug | EC_50_^a^ (μM) | FC^b^ | Determining cell |
| --- | --- | --- | --- | --- | --- |
| WT | WT | 9 | 0.13 ± 0.02 |  | TZM-bl |
|  |  | 9 | 0.10 ± 0.00 |  | PBMCs |
|  |  | 13 | 0.20 ± 0.02 |  | TZM-bl |
|  |  | 13 | 0.10 ± 0.01 |  | PBMCs |
|  |  | AZT | 0.11 ± 0.01 |  | TZM-bl |
|  |  | ABC | 12.24 ± 7.48 |  | TZM-bl |
|  |  | LMV | 1.23 ± 0.22 |  | TZM-bl |
|  |  | EFV | <1 nM |  | TZM-bl |
|  |  | NVP | 0.06 ± 0.00 |  | TZM-bl |
|  |  | RAL | <1 nM |  | TZM-bl |
|  |  | DTG | 1 nM |  | TZM-bl |
|  |  | NFV | <1 nM |  | PBMCs |
|  |  | DAV | <1 nM |  | PBMCs |
|  |  | SQV | 8.24 ± 0.15 nM |  | PBMCs |
| NRTI-resistant | 7396 | 9 | 0.07 ± 0.00 | 0.52 | TZM-bl |
|  |  | 13 | 0.15 ± 0.01 | 0.76 | TZM-bl |
|  |  | AZT | 16.42 ± 1.37 | 149.27 | TZM-bl |
|  |  | ABC | 22.41 ± 0.39 | 1.83 | TZM-bl |
|  |  | 3TC | 3.64 ± 1.37 | 2.96 | TZM-bl |
|  | 7406 | 9 | 0.09 ± 0.01 | 0.65 | TZM-bl |
|  |  | 13 | 0.16 ± 0.02 | 0.80 | TZM-bl |
|  |  | AZT | 73.12 ± 0.43 | >664.72 | TZM-bl |
|  |  | ABC | >100 | >8.17 | TZM-bl |
|  |  | 3TC | >100 | >81.3 | TZM-bl |
|  | 7407 | 9 | 0.07 ± 0.01 | 0.52 | TZM-bl |
|  |  | 13 | 0.19 ± 0.01 | 0.93 | TZM-bl |
|  |  | AZT | >100 | >909.09 | TZM-bl |
|  |  | ABC | >100 | >8.17 | TZM-bl |
|  |  | 3TC | >100 | >81.3 | TZM-bl |
| NNRTI- resistant | 12241 | 9 | 0.07 ± 0.00 | 0.56 | TZM-bl |
|  |  | 13 | 0.20 ± 0.00 | 0.99 | TZM-bl |
|  |  | EFV | 4.36 ± 0.08 | >1000 | TZM-bl |
|  |  | NVP | >100 | >1000 | TZM-bl |
|  | 12243 | 9 | 0.06 ± 0.00 | 0.49 | TZM-bl |
|  |  | 13 | 0.18 ± 0.01 | 0.90 | TZM-bl |
|  |  | EFV | 15.53 ± 3.39 | >1000 | TZM-bl |
|  |  | NVP | 23.91 ± 2.30 | 398.5 | TZM-bl |
| INSTI- resistant | 11847 | 9 | 0.07 ± 0.00 | 0.56 | TZM-bl |
|  |  | 13 | 0.14 ± 0.01 | 0.69 | TZM-bl |
|  |  | RAL | 4.99 ± 0.20 | >1000 | TZM-bl |
|  |  | DTG | 0.01 ± 0.01 | 13.75 | TZM-bl |
|  | 11849 | 9 | 0.08 ± 0.02 | 0.58 | TZM-bl |
|  |  | 13 | 0.23 ± 0.02 | 1.16 | TZM-bl |
|  |  | RAL | 14.32 ± 2.59 | >1000 | TZM-bl |
|  |  | DTG | 0.01 ± 0.00 | 9.92 | TZM-bl |
| PI- resistant | 11800 | 9 | 0.54 ± 0.25 | 5.4 | PBMCs |
|  |  |  | 0.08 ± 0.01 | 0.63 | TZM-bl |
|  |  | 13 | 0.81 ± 0.03 | 7.8 | PBMCs |
|  |  |  | 0.13 ± 0.10 | 0.64 | TZM-bl |
|  |  | NFV | 0.42 ± 0.02 | >1000 | PBMCs |
|  |  | DRV | 0.15 ± 0.02 | >1000 | PBMCs |
|  |  | SQV | 0.01 ± 0.00 | 0.68 | PBMCs |
|  | 11803 | 9 | 0.26 ± 0.08 | 2.6 | PBMCs |
|  |  |  | 0.07 ± 0.01 | 0.56 | TZM-bl |
|  |  | 13 | 0.28 ± 0.04 | 2.7 | PBMCs |
|  |  |  | 0.16 ± 0.02 | 0.80 | TZM-bl |
|  |  | NFV | 1.64 ± 0.22 | >1000 | PBMCs |
|  |  | DRV | 0.03 ± 0.01 | >1000 | PBMCs |
|  |  | SQV | 1.24 ± 0.13 | 150.93 | PBMCs |

^a^ The EC_50_ values were determined in TZM-bl or PBMCs. ^b^ Fold-change (FC) values were calculated by dividing the drug-resistant mutant HIV-1 EC_50_ by the WT EC_50_. EC_50_ and CC_50_ values in PBMCs were determined under a 10-fold serial dilution (0 ~ 100 μM) of compounds.
