# Supplementary material for: Discovery of new acetamide derivatives of 5-indole-1,3,4-oxadiazol-2-thiol as inhibitors of HIV-1 Tat-mediated viral transcription
Source: Antimicrob Agents Chemother. 2024 Sep 4;68(10):e00643-24. doi: 10.1128/aac.00643-24 (PMC11459959; doi:10.1128/aac.00643-24)
Supplement: Supplemental figures — Figures S1 to S6. [file aac.00643-24-s0002.pdf]

Supplementary Fig. S1

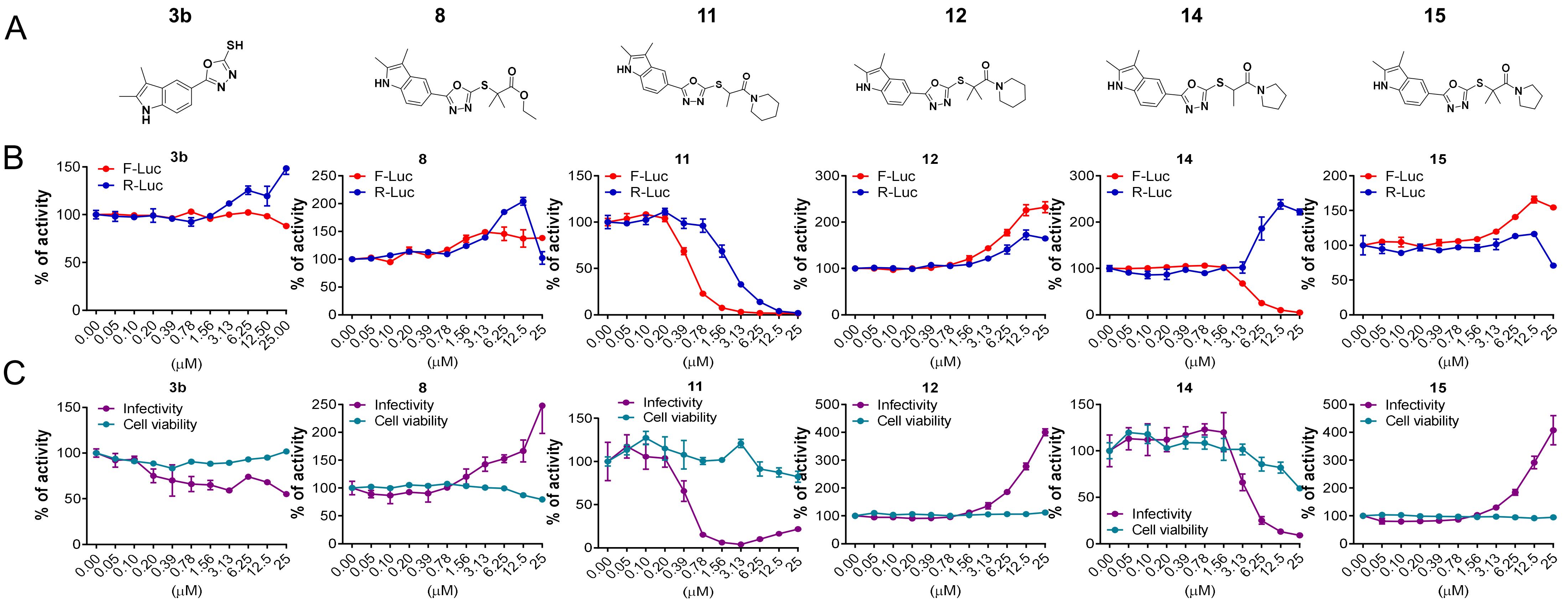

Supplementary Fig. S1. Continued

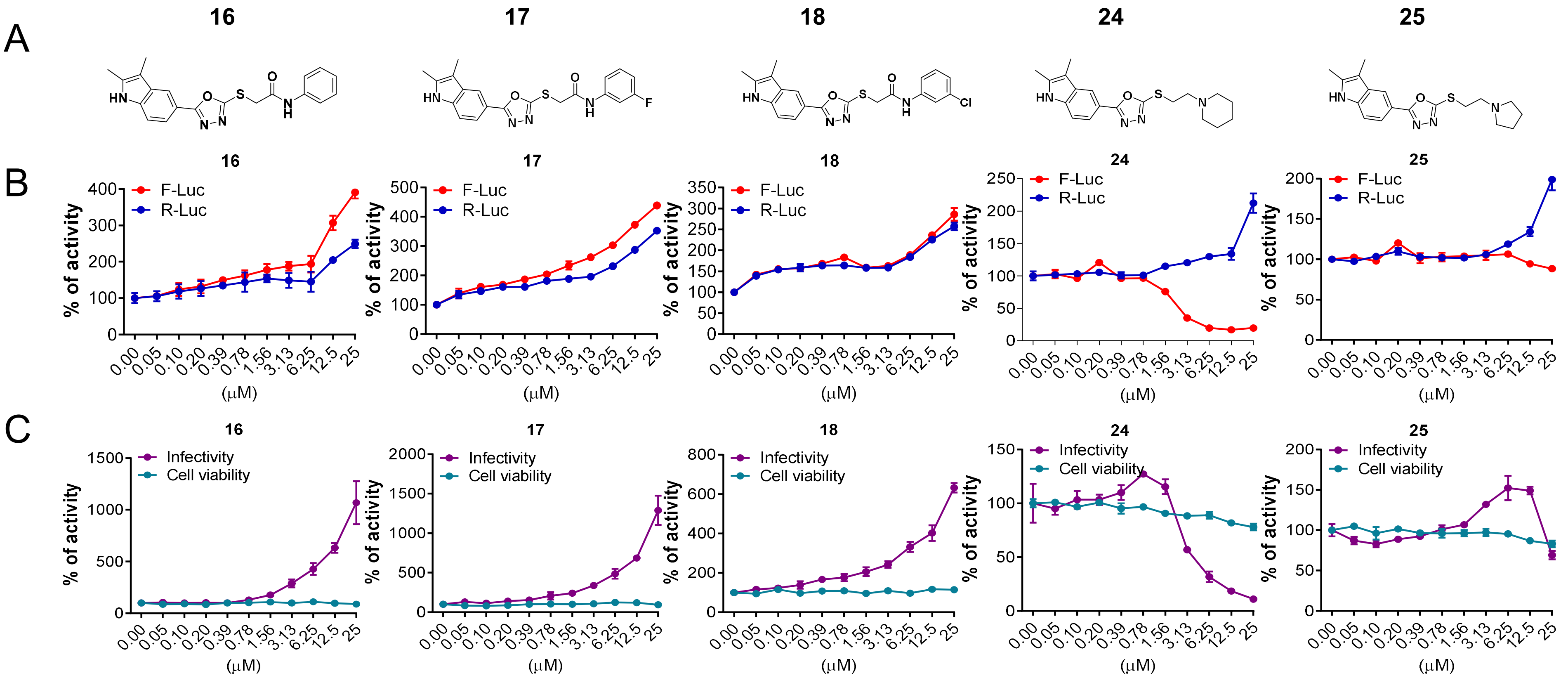

Supplementary Figure S1. A. Chemical structures of compounds. **B.** The bl-DTR cells (1×10^4) were treated with two-fold serial dilutions of each compound before adding doxycycline (Dox) (final concentration, 50 ng/mL). After 24 h of treatment, the activities of firefly luciferase (F-Luc, red line) and Renilla luciferase (R-Luc, black line) were determined using the Dual-Glo™ Luciferase assay kit. **C.** TZM-bl cells were treated with the indicated compound and subsequently infected with the HIV-1_{NL4-3} strain at a multiplicity of infection (MOI) of 1. At 48 h after infection, the viral infectivity (purple line) and cell viability (black line) were determined using the Bright-Glo™ luciferase assay kit and PrestoBlue Cell Viability Reagent™, respectively. The relative activities are represented as the mean ± SD (n = 3) compared with those of the vehicle (DMSO, 0 μM).

Supplementary Fig. S2

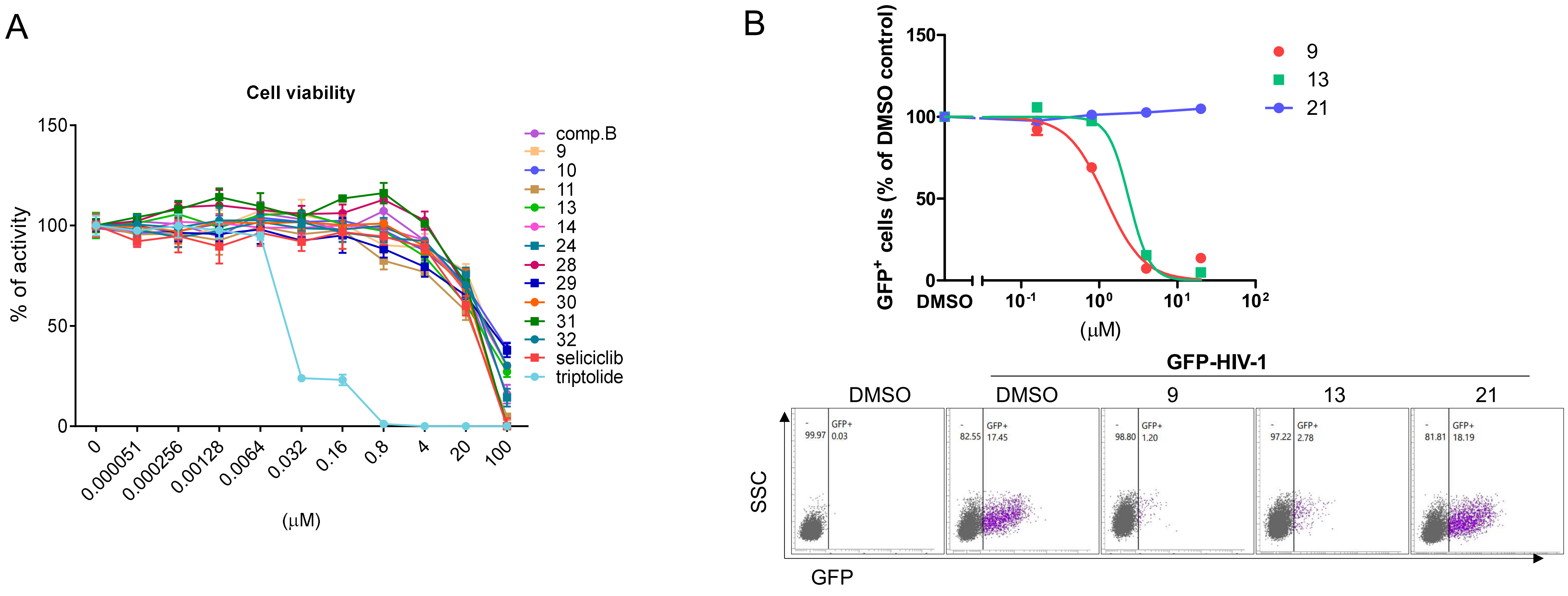

Supplementary Figure S2. A. TZM-bl (1×10^4) were treated with five-fold serial dilutions of each compound. At 48 h after treatment, cell viability was analyzed with resazurin-based PrestoBlue Cell Viability Reagent as described in Materials and Methods. **B.** TZM-bl cells (7×10^4) were treated with five-fold serial dilutions of each compound and then infected with the HIV-$1_{NL4\text{-}3}$IRES-eGFP-nef$^+$ strain at an MOI of 0.5. At 48 h after infection, the eGFP-expressing cell counts were determined using flow cytometry. The upper graphical data represent the relative percentages compared with that of the vehicle (DMSO, 0 μM).) Bottom panel shows FACS data for 4 μM compound treatment.

Supplementary Fig. S3

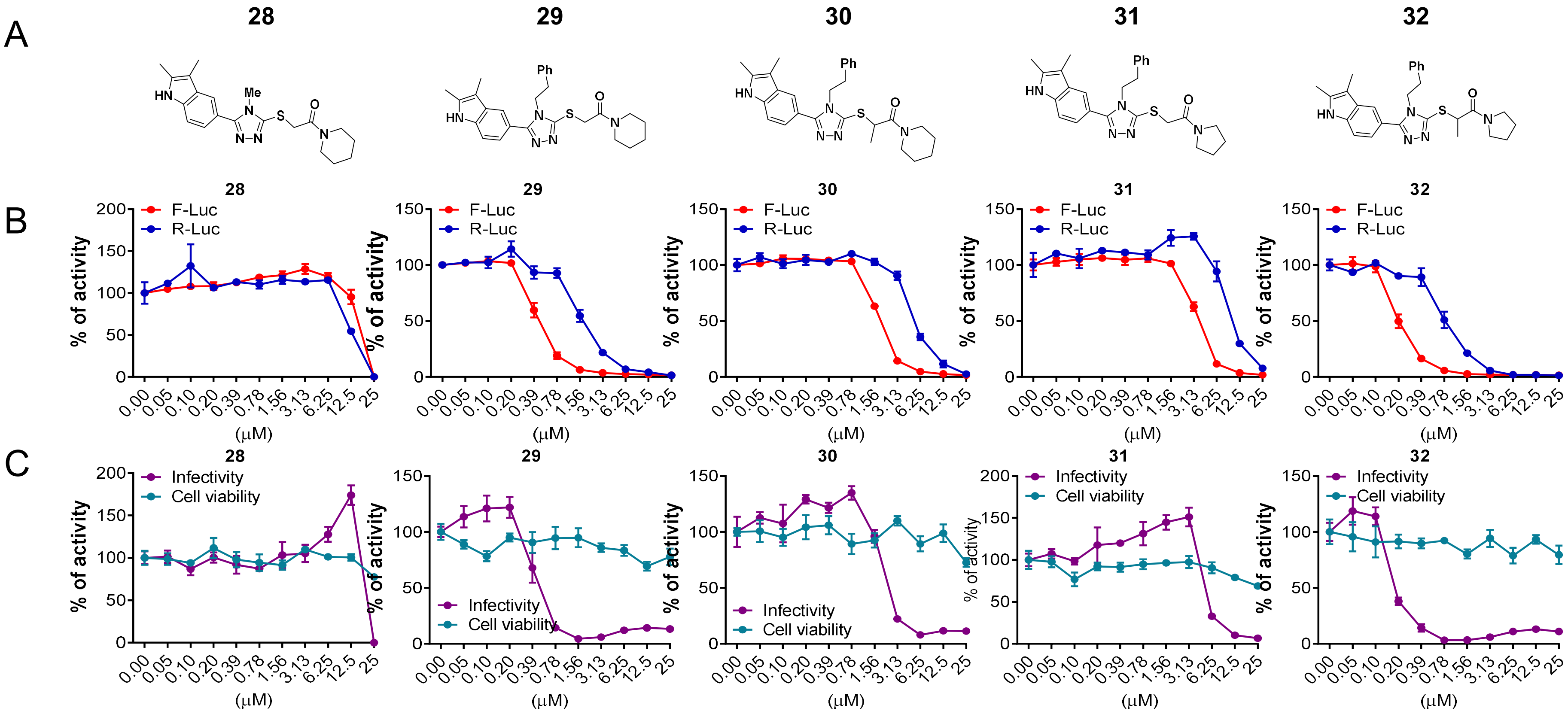

Supplementary Figure S3. A. Chemical structures of compounds (Me: methyl; Ph: phenyl). **B.** The bl-DTR cells (1 × 10^4) were treated with two-fold serial dilutions of each compound before adding Dox (final concentration, 50 ng/mL). After 24 h of treatment, the activities of F-Luc (red line) and R-Luc (black line) were determined using the Dual-Glo^{TM} Luciferase assay kit. **C.** The TZM-bl cells were treated with the indicated compounds and then infected with the HIV-1_{NL4-3} strain at an MOI of 1. At 48 h after infection, the viral infectivity (purple line) and cell viability (black line) were determined using the Bright-Glo^{TM} luciferase assay kit and PrestoBlue Cell Viability Reagent^{TM}, respectively. The relative activities are represented as the mean ± SD (n = 3) compared with those of the vehicle (DMSO, 0 μM).

Supplementary Fig. S4

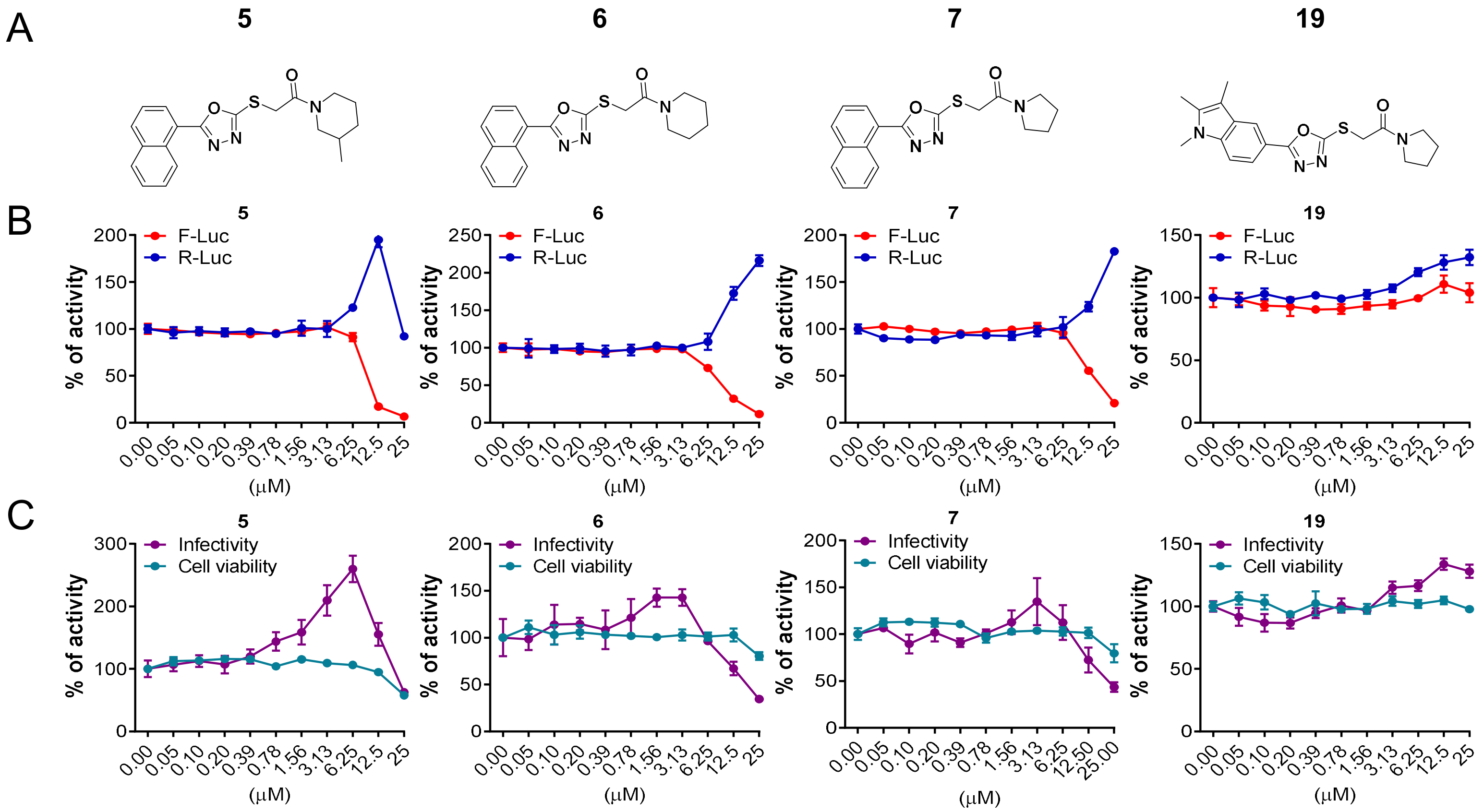

Supplementary Figure S4. A. Chemical structures of compounds. **B.** The bl-DTR cells (1 × 10^4) were treated with two-fold serial dilutions of each compound before adding Dox (final concentration, 50 ng/mL). After 24 h of treatment, the activities of F-Luc (red line) and R-Luc (black line) were determined using the Dual-Glo™ Luciferase assay kit. **C.** The TZM-bl cells were treated with the indicated compounds and then infected with the HIV-1_{NL4-3} strain at an MOI of 1. At 48 h after infection, the viral infectivity (purple line) and cell viability (black line) were determined using the Bright-Glo™ luciferase assay kit and PrestoBlue Cell Viability Reagent™, respectively. The relative activities are represented as the mean ± SD (n = 3) compared with those of the vehicle (DMSO, 0 μM).

Supplementary Fig. S5

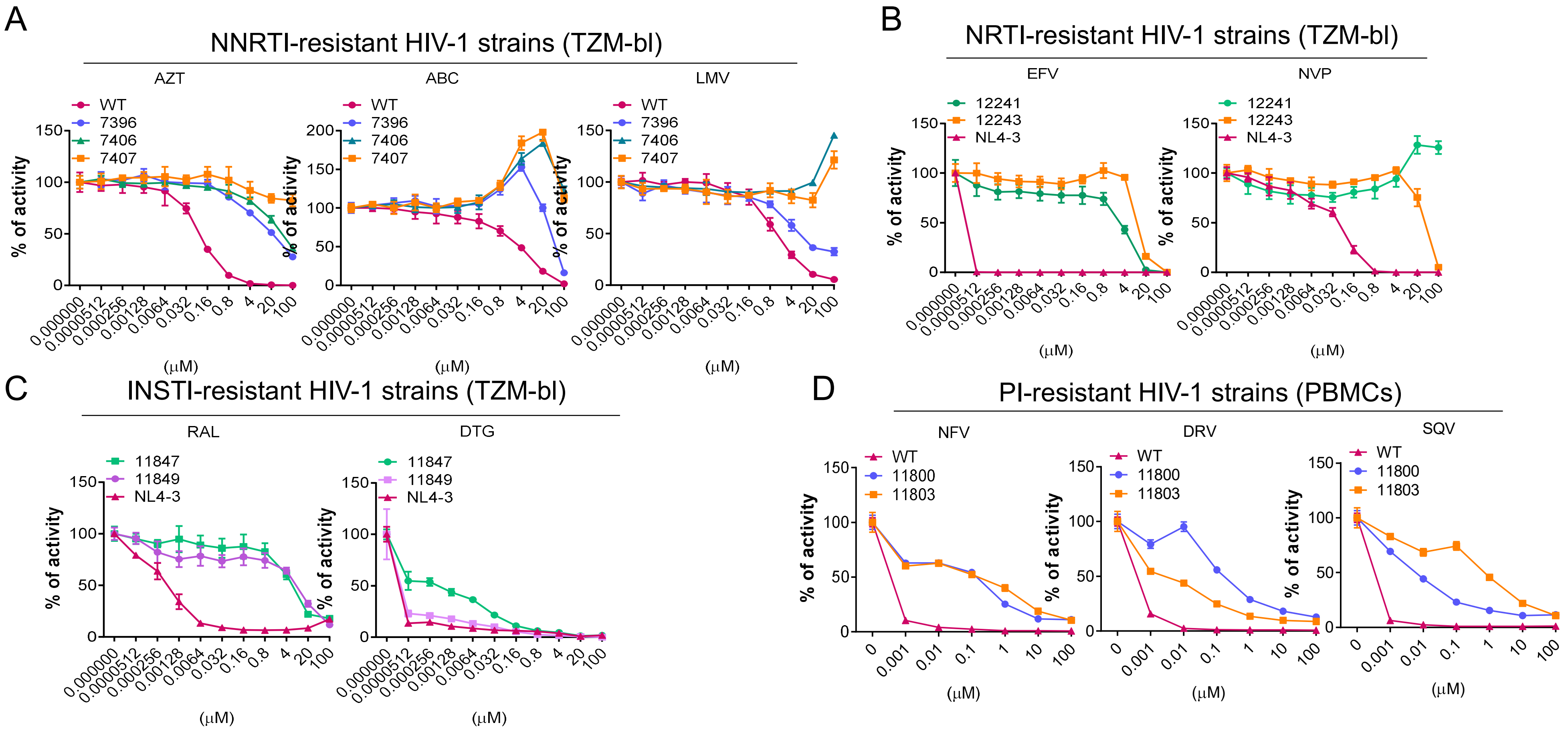

Supplementary Figure S5. A-C. The TZM-bl cells (1 × 10^4 cells/well) cultured in 96-well plates were treated with five-fold serial dilutions of the indicated compounds for 1 h prior to infection with the HIV-1_{NL4-3} and ARVs-resistant HIV-1 strains at an MOI of 1. At 48 h after infection, viral infectivity was determined using an F-Luc assay kit. **D**. Peripheral blood mononuclear cells (PBMCs) (4 × 10^5) cells/well) were infected with the HIV-1_{NL4-3} and protease inhibitor (PI)-resistant HIV-1 strains at an MOI of 0.1. Subsequently, PBMCs were treated with 10-fold serial dilutions of the indicated PIs. Three days after infection, the inhibitory effect of PIs on viral replication was determined using the p24 ALPHALISA assay kit. The relative activities are represented as the mean ± SD (n = 3) compared with those of the vehicle (DMSO, 0 μM).

Supplementary Fig. S6

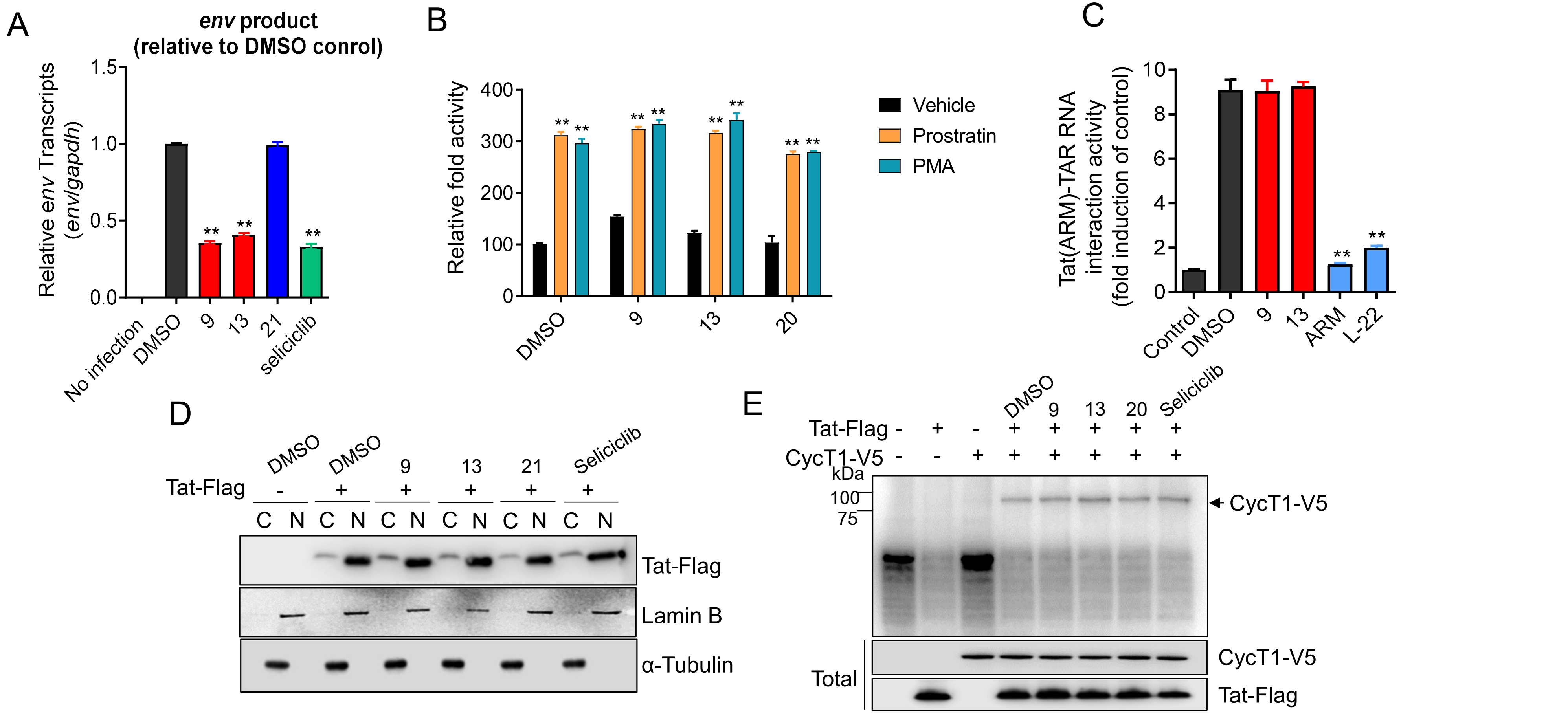

Supplementary Figure S6. A. Quantitative real-time PCR of HIV-1 mRNA from HIV-1_{NL4-3}-infected A3.01 cells treated or untreated with the indicated compounds was performed using the *env* primer set, and levels were normalized to those of *gapdh*. The data represent the relative fold change compared with DMSO treatment (n = 3). **B.** Effect of the compounds on NF-κB promoter activity assessed using a luciferase assay. HeLa cells (2.5×10^5) were transfected with NF-κB RE-driven F-Luc plasmid (1 μg) and pCMV-R-Luc (200 ng). After 24 h, 10 μM of each compound was administered with phorbol 12-myristate 13-acetate (PMA) (50 ng/mL) or prostratin (10 μM) for 24 h. The luciferase activity was determined using a Dual-Glo^{TM} Luciferase assay kit (Promega). The data normalized to the R-Luc activity are expressed as the mean ± SD (n = 3) of the relative fold activity compared with the no-PMA (vehicle) treatment. **C**. Time-resolved fluorescence resonance energy transfer (TR-FRET) assay for the Tat (arginine-rich motif [ARM]) and transactivating response element (TAR) RNA interaction. Wild-type Flag-tagged ARM peptide (50 nmol) was incubated with 5′-Cy5-TAR RNA in the presence of the indicated compounds or known inhibitors (ARM peptide, L-22). Subsequently, europium-conjugated α-Flag antibody was added to the reaction and incubated for 30 min, and the interaction signal was detected on an EnSight plate reader (PerkinElmer). The data are expressed as the mean ± SD (n = 3) of relative fold activity compared with the control lacking Flag-ARM. **D.** Levels of Tat protein in fractions of the nucleus (N) and cytoplasm (C). HEK293T cells were transfected with Flag-Tat86-expressing plasmid 24 h prior to treatment with the indicated compounds (1.5 μM). One day later, the cells were fractionated using a cell fractionation kit (Pierce). Tat protein levels were assessed through western blotting using antibodies against Flag, lamin B (an indicator of the nuclear fraction), and tubulin (an indicator of the cytosolic fraction). **E.** Co-immunoprecipitation of Tat and cyclin T1. HeLa cells were co-transfected with Flag-Tat86 and V5-Cyclin T1 expression plasmids and cultured in the presence of the indicated compounds. The cells were harvested and immunoprecipitated using an anti-Flag antibody. Co-immunoprecipitated cyclin T1 (CycT1)-V5 was detected by western blotting using an anti-V5 antibody. (A-C) ** $P < 0.01$ compared with the vehicle (DMSO).
